# Precision analysis model and experimentation of vision reconstruction with two cameras and 3D orientation reference

**DOI:** 10.1038/s41598-021-83390-y

**Published:** 2021-02-16

**Authors:** Rong Chen, Fang Chen, Guan Xu, Xiaotao Li, Hui Shen, Jing Yuan

**Affiliations:** 1 Transportation College, Nanling Campus, Jilin University, Renmin Str. 5988#, Changchun, China; 2 School of Mechanical and Aerospace Engineering, Nanling Campus, Jilin University, Renmin Str. 5988#, Changchun, China

**Keywords:** Imaging and sensing, Optical metrology

## Abstract

Active vision reconstruction is widely used in industrial manufacturing and three-dimensional inspection. The reconstruction accuracy is an important problem to be investigated for the inspection process. The paper conducts an analysis study of the reconstruction error for the vision reconstruction with a planar laser, two cameras and a 3D orientation board. The variation principles of the spatial coordinates caused by the variations of the extrinsic parameters of the cameras, intrinsic parameters of the internal camera, and image coordinate points of the internal camera, are modeled and analyzed in this paper. The analysis is also proved by the verification experiments, which provides the application potential for other active-vision-based reconstructions.

## Introduction

3D reconstruction has been considered as an important approach to acquire the necessary information for the mobile robot^1^, industrial inspection^2,3^, medical image^4,5^, industrial manufacture^6^, vehicle navigation^7^, and pattern recognition^8–10^. The spatial reconstruction initially captures the image of the measured object and then transfers 2D information to 3D information by the additional conditions.

The binocular vision is a reconstruction method using two or more cameras with the overlapping view fields. The second camera contributes a projection relationship and complements two degrees of the unknown 3D point. Stelzer et al.^11^ present a navigation method for the robot with six legs, which walks on the sand surface. In the navigation, the binocular vision images are employed to construct the depth map, which is solved by the semi-global matching algorithm. The visual odometry and inertial information are computed for the pose estimation. Lee et al.^12^ propose a vehicle detection algorithm with the stereo vision technology. The pavement characteristic and disparity histogram are employed to recognize the vehicle in the complicated traffic situations, especially the obstacle situation. The obstacle positioning, segmentation and vehicle recognition are the components of the algorithm. Correal et al.^13^ present a terrain reconstruction method with stereo images to achieve the autonomous navigation of the robots in complex environment. The information of the inertial measurement unit is fused in the robot navigation system. The stereo pair is refined by the adjustment of a function. Then, the pair set is computed by the disparity and the terrain is reconstructed by the re-projection operation. Chen et al.^14^ establish a multi-view measurement system to increase the observation range in optical reconstructions. The local calibration and global calibration are realized to unify the point clouds from the cameras. A point cloud matching method is designed to generate the global cloud and improve the precision of the reconstruction. Tang et al.^15^ propose a vision system with four cameras to achieve the 3D reconstruction of large-scale steel tubular structures. The point cloud is generated and filtered by the four-camera system. A deep learning algorithm of geometrical features is performed to matching the point clouds. The reconstruction diameters of the tested objects are considered as the benchmark, which proves the validity and performance of the reconstruction method. Chen et al.^16^ provides a 3D perception approach of orchard banana central stock with the adaptive multi-vision system. The semantic segmentation network is trained to realize the stereo matching that ensures the 3D triangulation for different positions of the system. The reconstruction of the stereo or binocular vision depends on the feature matching, which is often effective for the rough terrain and unavailable for the lubricous surface without feature points. The active vision recoveries the 3D point from the image by the active light mark which is generated from the laser-projection on the object or the coded-light-projection on the object from the DLP projector. Zhang et al.^17^ introduce the inspection method based on a cross-light projector and a camera. The laser stripe of the cross-light on the weld is captured by the camera and extracted from the image. A planar target is designed to calibrate the system. Xu et al.^18^ detect the 3D surface of the vehicle part by the active vision. A structured monochromatic light with the one-shot pattern is proposed to reconstruct the 3D area. The benefits of the method include the availability in the ambient illumination and the part with the reflective characteristic. Yee et al.^19^ outline a profilometry method with the color-sinusoidal-structured-light to simplify the fringe analysis. The direct arccosine function and De Bruijn sequence are adopted to demodulate the fringe images. Newcombe et al.^20^ realize the real-time 3D reconstruction based on RGB-D camera for the first time. In this paper, the model of the truncated signed distance function is used to continuously fuse the depth image and reconstruct the 3D surface. In addition, it is more accurate to calculate the pose by registration of the current frame and the image of the model projection than by the registration of the current frame and the previous frame. Xu et al.^21^ introduce a reconstruction system with a laser plane, a 3D moveable board, a camera inside the board and an external camera. The 3D moveable reference constructs the Cartesian coordinate system. As the external camera observes the board and the internal camera obtains the image of the intersection between the laser plane and the object, the measurement system is more flexible than the fixed one. The structured-light-based reconstruction actively marks the light label on the object. Thus, it is reliable in the industry and the environment with the complicated illumination field.

The 3D reconstruction with the camera and laser is based on the triangulation. Therefore, the precision of the measurement system relies on the system structure parameters. Llorca et al.^22^ present an error estimation approach for the stereo vision system to detect the pedestrian ahead of the vehicle. The pedestrian is inspected by the 3D cluster method and support vector machine (SVM). The parameters of the sensors are analyzed to study the influence on the reconstruction errors. The two corresponding points on the two cameras are unified to a vector to analyze the 3D point error. The influences of the focal length and the baseline are also studied in the paper. Belhaoua et al.^23^ analyze the reconstruction errors of the stereo-vision-based system. The analysis of the edge detection of straight line segments and elliptical arcs is performed in the reconstruction process. The singular value decomposition is adopted to estimate the parameters of the fitting lines. The perpendicular distances from the pixels to the fitting line stand for the errors. The uncertainties of the 3D reconstruction from the feature extraction process are estimated by the stereo vision model. Sankowski et al.^24^ introduce a computation model and formulas to estimate the precision of the binocular vision system. The method only requires a 2D calibration board and a laser distance meter. Yang et al.^25^ study the errors of the system structure parameters of the stereo vision and the correlation model is also analyzed for the system. Jiang et al.^26^ investigate the accuracy of 3D reconstruction from the Kinect-based RGB-D camera. In the method, a CAD model from a simulation program is adopted to generate the depth and pose data, which is considered as the benchmark of the evaluation. The reconstruction model is introduced in the paper and realized by a 6-DOF robot manipulator. The previous studies focus on the analysis of the pose and parameters of the monocular vision system, stereo vision system, and single-camera-based active vision system. However, the reconstruction system in Ref.^21^ consists of a laser plane, a 3D orientation board, an internal camera inside the reference and an external camera. In the reconstruction of the system, the external camera only observes the 3D cubic board and generates the 3D homography of the board. Then the internal camera inside the board captures the projection of the laser plane on the measured object. The object outside the view field of the external camera can be reconstructed by the homograph of the cubic board, as the cubic board freely moves in the view field of the external camera. Thus, the vision system provides a large view scope, especially for the area that cannot be directly observed by the external camera. The reconstruction system is a chained-form system including a laser plane, a 3D orientation board, an internal camera inside the reference and an external camera. The different poses among the above instrumentations conduct different reconstruction errors in a transfer chain. Thus, we investigate the chained-form transmission of the errors generated from the system structure parameters and camera parameters by simulations and experiments.

The rest paper includes three sections. Section “Analysis model” introduces the reconstruction system in Ref.^21^ and constructs the analysis model for the intrinsic parameters and extrinsic parameters. Section “Results of precision analysis and experimentation” demonstrates the simulations and experiments of the analysis results. Section “Summary” concludes the paper.

## Analysis model

**Figure 1:**
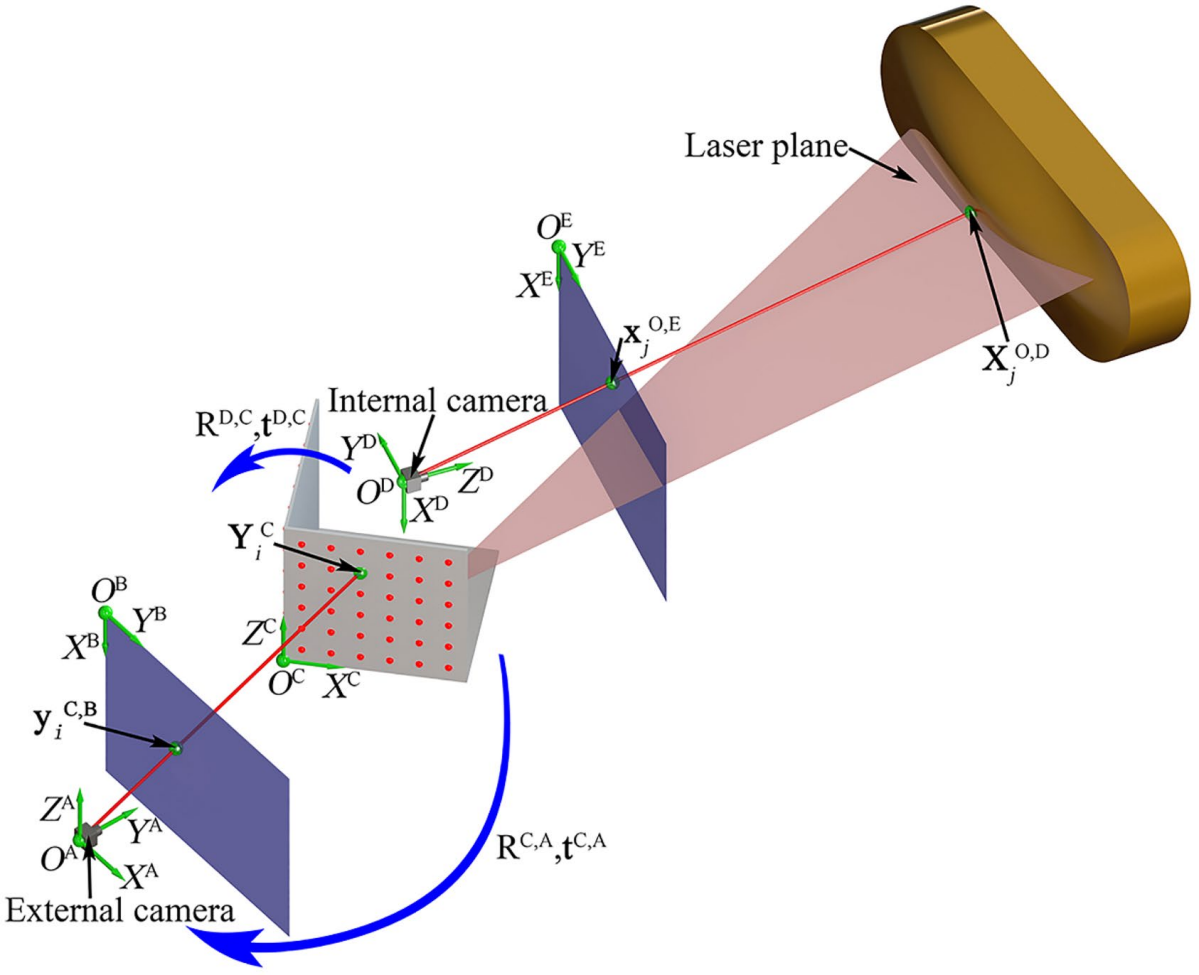
The principle of the vision reconstruction with the laser plane, internal camera, 3D reference and external camera in Ref.^21^. The planar laser intersects the measured object with the 3D point ${\mathbf{X}}_{j}^{{\text{O,D}}}$ in CSIC. Then the 3D point ${\mathbf{X}}_{j}^{{\text{O,D}}}$ in CSIC is transformed to the 3D laser point ${\mathbf{X}}_{j}^{{\text{C}}}$ in CSOB. Finally, the 3D point is represented by ${\mathbf{X}}_{j}^{{\text{A}}}$ in CSEC.

The active vision system in Ref.^21^ is established to provide the spatial coordinate of the object point. For the reconstruction purpose, the measurement system consists of a planar laser emitter, a 3D orientation board covered by the LED array, a camera inside the orientation board, and a camera outside the orientation board. The internal camera captures the image of the laser projection point on the object. The orientation board is placed in the view field of the external camera. The external camera only contributes the orientation board image, which does not contain the projection laser point on the object. The reconstruction process is exhibited in Fig. 1. The extrinsic parameters and the intrinsic parameters of the reconstruction instrumentation are calibrated by the calibration object and the calibration details are explained in Ref.^21^. Here, we employ the method in Ref.^25^ to represent the variations by the derivatives of the point coordinates. However, the system studied in Ref.^25^ is a stereo vision system, rather than a chained-form active vision system with a planar laser, a 3D moveable board, an internal camera inside the orientation board and an external camera. *O*^E^-*X*^E^*Y*^E^, *O*^D^-*X*^D^*Y*^D^*Z*^D^, *O*^C^-*X*^C^*Y*^C^*Z*^C^, *O*^B^-*X*^B^*Y*^B^ and *O*^A^-*X*^A^*Y*^A^*Z*^A^ stand for the coordinate systems of the internal-camera-image (CSICI), the orientation board (CSOB), the external-camera-image (CSECI), the external camera (CSEC), and the internal camera (CSIC), individually. The study assumes that the random variables of the system parameters are independent and identical distributed.

The basic measurement model of the vision system can be concluded by three steps^21^. In the first step, the planar laser intersects the measured object with the 3D point ${\mathbf{X}}_{j}^{{\text{O,D}}} = \left[ {X_{j}^{{\text{O,D}}} ,Y_{j}^{{\text{O,D}}} ,Z_{j}^{{\text{O,D}}} ,1} \right]^{{\text{T}}}$ in CSIC. According to the conditions of the point-projection and the point on the laser plane, ${\mathbf{X}}_{j}^{{\text{O,D}}}$ satisfies^27,28^

1 $${\text{K}}^{{\text{D}}} \left[ {\begin{array}{*{20}l} {X_{j}^{{\text{O,D}}} } \hfill \\ {Y_{j}^{{\text{O,D}}} } \hfill \\ {Z_{j}^{{\text{O,D}}} } \hfill \\ \end{array} } \right] = {\mathbf{x}}_{j}^{{\text{O,E}}}$$

2 $$({\mathbf{\Pi}}^{{\text{D}}} )^{{\text{T}}} {\mathbf{X}}_{j}^{{\text{O,D}}} = 0,$$

where ${\mathbf{x}}_{j}^{{{\text{O}} ,{\text{E}}}} = \left[ {x_{j}^{{{\text{O}} ,{\text{E}}}} ,y_{j}^{{{\text{O}} ,{\text{E}}}} ,1} \right]^{\text{T}}$ is the projection of ${\mathbf{X}}_{j}^{{\text{O,D}}}$, ${\mathbf{\Pi}}^{{\text{D}}} = [\pi_{1} ,\pi_{2} ,\pi_{3} ,\pi_{4} ]^{{\text{T}}}$ is the planar laser coordinate, $${\text{K}}^{{\text{D}}} = \left[ {\begin{array}{*{20}c} {\alpha^{{\text{D}}} } & {\gamma^{{\text{D}}} } & {u^{{\text{D}}} } \\ 0 & {\beta^{{\text{D}}} } & {v^{{\text{D}}} } \\ 0 & 0 & 1 \\ \end{array} } \right]$$ is the intrinsic parameter matrix of the internal camera. The 3D laser point ${\mathbf{X}}_{j}^{{{\text{O}},{\text{D}}}}$ in CSIC can be solved by Eqs. (1) and (2).

In the second step, the spatial coordinate of the laser point on the object is transformed to CSOB by

3 $${\mathbf{X}}_{j}^{{\text{C}}} = \left[ {\begin{array}{*{20}c} {{\text{R}}^{{\text{D,C}}} } & {{\mathbf{t}}^{{\text{D,C}}} } \\ {{\mathbf{0}}_{3 \times 1}^{\text{T}} } & 1 \\ \end{array} } \right]{\mathbf{X}}_{j}^{{\text{O,D}}},$$

where ${\mathbf{X}}_{j}^{{{\text{O}},{\text{D}}}}$ and ${\mathbf{X}}_{j}^{{\text{C}}}$ are the spatial coordinate of the laser point in CSIC and CSOB, respectively. $${\text{R}}^{{\text{D,C}}} = \left[ {\begin{array}{*{20}c} {r_{11}^{{\text{D,C}}} } & {r_{12}^{{\text{D,C}}} } & {r_{13}^{{\text{D,C}}} } \\ {r_{21}^{{\text{D,C}}} } & {r_{22}^{{\text{D,C}}} } & {r_{23}^{{\text{D,C}}} } \\ {r_{31}^{{\text{D,C}}} } & {r_{32}^{{\text{D,C}}} } & {r_{33}^{{\text{D,C}}} } \\ \end{array} } \right]$$ and $${\mathbf{t}}^{{\text{D,C}}} = \left[ {\begin{array}{*{20}c} {t_{x}^{{\text{D,C}}} } & {t_{y}^{{\text{D,C}}} } & {t_{z}^{{\text{D,C}}} } \\ \end{array} } \right]^{{\text{T}}}$$ are the rotation and the translation from CSIC to CSOB.

In the third step, the spatial coordinate of the laser point in CSOB is transformed to CSEC by

4 $${\mathbf{X}}_{j}^{{\text{A}}} = \left[ {\begin{array}{*{20}c} {{\text{R}}^{{\text{C,A}}} } & {{\mathbf{t}}^{{\text{C,A}}} } \\ {{\mathbf{0}}_{3 \times 1}^{\text{T}} } & 1 \\ \end{array} } \right]{\mathbf{X}}_{j}^{{\text{C}}},$$

where ${\mathbf{X}}_{j}^{{\text{A}}}$ is the spatial coordinate of the laser point in CSEC, $${\text{R}}^{{\text{C,A}}} = \left[ {\begin{array}{*{20}c} {r_{11}^{{\text{C,A}}} } & {r_{12}^{{\text{C,A}}} } & {r_{13}^{{\text{C,A}}} } \\ {r_{21}^{{\text{C,A}}} } & {r_{22}^{{\text{C,A}}} } & {r_{23}^{{\text{C,A}}} } \\ {r_{31}^{{\text{C,A}}} } & {r_{32}^{{\text{C,A}}} } & {r_{33}^{{\text{C,A}}} } \\ \end{array} } \right]$$ and $${\mathbf{t}}^{{\text{C,A}}} = \left[ {\begin{array}{*{20}c} {t_{x}^{{\text{C,A}}} } & {t_{y}^{{\text{C,A}}} } & {t_{z}^{{\text{C,A}}} } \\ \end{array} } \right]^{{\text{T}}}$$ are the rotation and the translation from CSOB to CSEC.

According to Eqs. (3) and (4), the 3D laser point ${\mathbf{X}}_{j}^{{\text{A}}}$ in CSEC is

5 $${\mathbf{X}}_{j}^{{\text{A}}} = {\text{H}}^{{\text{D,A}}} {\mathbf{X}}_{j}^{{\text{O,D}}},$$

where the homography $${\text{H}}^{{\text{D,A}}} = \left[ {\begin{array}{*{20}c} {h_{11}^{{\text{D,A}}} } & {h_{12}^{{\text{D,A}}} } & {h_{13}^{{\text{D,A}}} } & {h_{14}^{{\text{D,A}}} } \\ {h_{21}^{{\text{D,A}}} } & {h_{22}^{{\text{D,A}}} } & {h_{23}^{{\text{D,A}}} } & {h_{24}^{{\text{D,A}}} } \\ {h_{31}^{{\text{D,A}}} } & {h_{32}^{{\text{D,A}}} } & {h_{33}^{{\text{D,A}}} } & {h_{34}^{{\text{D,A}}} } \\ 0 & 0 & 0 & 1 \\ \end{array} } \right]$$, $h_{11}^{{\text{D,A}}} = r_{11}^{{\text{C,A}}} r_{11}^{{\text{D,C}}} + r_{12}^{{\text{C,A}}} r_{21}^{{\text{D,C}}} + r_{13}^{{\text{C,A}}} r_{31}^{{\text{D,C}}}$, $h_{12}^{{\text{D,A}}} = r_{11}^{{\text{C,A}}} r_{12}^{{\text{D,C}}} + r_{12}^{{\text{C,A}}} r_{22}^{{\text{D,C}}} + r_{13}^{{\text{C,A}}} r_{32}^{{\text{D,C}}}$,

$$h_{13}^{{\text{D,A}}} = r_{11}^{{\text{C,A}}} r_{13}^{{\text{D,C}}} + r_{12}^{{\text{C,A}}} r_{23}^{{\text{D,C}}} + r_{13}^{{\text{C,A}}} r_{33}^{{\text{D,C}}}$$, $$h_{14}^{{\text{D,A}}} = r_{11}^{{\text{C,A}}} t_{x}^{{\text{D,C}}} + r_{12}^{{\text{C,A}}} t_{y}^{{\text{D,C}}} + r_{13}^{{\text{C,A}}} t_{z}^{{\text{D,C}}} + t_{x}^{{\text{C,A}}}$$,

$$h_{21}^{{\text{D,A}}} = r_{21}^{{\text{C,A}}} r_{11}^{{\text{D,C}}} + r_{22}^{{\text{C,A}}} r_{21}^{{\text{D,C}}} + r_{23}^{{\text{C,A}}} r_{31}^{{\text{D,C}}}$$, $$h_{22}^{{\text{D,A}}} = r_{21}^{{\text{C,A}}} r_{12}^{{\text{D,C}}} + r_{22}^{{\text{C,A}}} r_{22}^{{\text{D,C}}} + r_{23}^{{\text{C,A}}} r_{32}^{{\text{D,C}}}$$,

$$h_{23}^{{\text{D,A}}} = r_{21}^{{\text{C,A}}} r_{13}^{{\text{D,C}}} + r_{22}^{{\text{C,A}}} r_{23}^{{\text{D,C}}} + r_{23}^{{\text{C,A}}} r_{33}^{{\text{D,C}}}$$, $$h_{24}^{{\text{D,A}}} = r_{21}^{{\text{C,A}}} t_{x}^{{\text{D,C}}} + r_{22}^{{\text{C,A}}} t_{y}^{{\text{D,C}}} + r_{23}^{{\text{C,A}}} t_{z}^{{\text{D,C}}} + t_{y}^{{\text{C,A}}}$$,

$$h_{31}^{{\text{D,A}}} = r_{31}^{{\text{C,A}}} r_{11}^{{\text{D,C}}} + r_{32}^{{\text{C,A}}} r_{21}^{{\text{D,C}}} + r_{33}^{{\text{C,A}}} r_{31}^{{\text{D,C}}}$$, $$h_{32}^{{\text{D,A}}} = r_{31}^{{\text{C,A}}} r_{12}^{{\text{D,C}}} + r_{32}^{{\text{C,A}}} r_{22}^{{\text{D,C}}} + r_{33}^{{\text{C,A}}} r_{32}^{{\text{D,C}}}$$,

$$h_{33}^{{\text{D,A}}} = r_{31}^{{\text{C,A}}} r_{13}^{{\text{D,C}}} + r_{32}^{{\text{C,A}}} r_{23}^{{\text{D,C}}} + r_{33}^{{\text{C,A}}} r_{33}^{{\text{D,C}}}$$, $$h_{34}^{{\text{D,A}}} = r_{31}^{{\text{C,A}}} t_{x}^{{\text{D,C}}} + r_{32}^{{\text{C,A}}} t_{y}^{{\text{D,C}}} + r_{33}^{{\text{C,A}}} t_{z}^{{\text{D,C}}} + t_{z}^{{\text{C,A}}}$$.

Stacking Eqs. (1)–(5), the coordinates of $${\mathbf{X}}_{j}^{{\text{A}}} = \left[ {\begin{array}{*{20}c} {X_{j}^{{\text{A}}} } & {Y_{j}^{{\text{A}}} } & {Z_{j}^{{\text{A}}} } & 1 \\ \end{array} } \right]^{{\text{T}}}$$ in CSEC are

6 $$X_{j}^{{\text{A}}} = \pi_{4} \frac{{\left( {h_{11}^{{\text{D,A}}} - h_{13}^{{\text{D,A}}} \frac{{\pi_{1} }}{{\pi_{3} }}} \right)\left[ {\beta^{{\text{D}}} (u^{{\text{D}}} - x_{j}^{{{\text{O}} ,{\text{E}}}} ) - \gamma^{{\text{D}}} (v^{{\text{D}}} - y_{j}^{{{\text{O}} ,{\text{E}}}} )} \right] + \alpha^{{\text{D}}} (v^{{\text{D}}} - y_{j}^{{{\text{O}} ,{\text{E}}}} )\left( {h_{12}^{{\text{D,A}}} - h_{13}^{{\text{D,A}}} \frac{{\pi_{2} }}{{\pi_{3} }}} \right)}}{{\alpha^{{\text{D}}} \beta^{{\text{D}}} \pi_{3} - \pi_{1} \left[ {\beta^{{\text{D}}} (u^{{\text{D}}} - x_{j}^{{{\text{O}} ,{\text{E}}}} ) - \gamma^{{\text{D}}} (v^{{\text{D}}} - y_{j}^{{{\text{O}} ,{\text{E}}}} )} \right] - \pi_{2} \alpha^{{\text{D}}} (v^{{\text{D}}} - y_{j}^{{{\text{O}} ,{\text{E}}}} )}} - h_{13}^{{\text{D,A}}} \frac{{\pi_{4} }}{{\pi_{3} }} + h_{14}^{{\text{D,A}}}$$

7 $$Y_{j}^{{\text{A}}} = \pi_{4} \frac{{\left( {h_{21}^{{\text{D,A}}} - h_{23}^{{\text{D,A}}} \frac{{\pi_{1} }}{{\pi_{3} }}} \right)\left[ {\beta^{{\text{D}}} (u^{{\text{D}}} - x_{j}^{{{\text{O}} ,{\text{E}}}} ) - \gamma^{{\text{D}}} (v^{{\text{D}}} - y_{j}^{{{\text{O}} ,{\text{E}}}} )} \right] + \alpha^{{\text{D}}} (v^{{\text{D}}} - y_{j}^{{{\text{O}} ,{\text{E}}}} )\left( {h_{22}^{{\text{D,A}}} - h_{23}^{{\text{D,A}}} \frac{{\pi_{2} }}{{\pi_{3} }}} \right)}}{{\alpha^{{\text{D}}} \beta^{{\text{D}}} \pi_{3} - \pi_{1} \left[ {\beta^{{\text{D}}} (u^{{\text{D}}} - x_{j}^{{{\text{O}} ,{\text{E}}}} ) - \gamma^{{\text{D}}} (v^{{\text{D}}} - y_{j}^{{{\text{O}} ,{\text{E}}}} )} \right] - \pi_{2} \alpha^{{\text{D}}} (v^{{\text{D}}} - y_{j}^{{{\text{O}} ,{\text{E}}}} )}} - h_{23}^{{\text{D,A}}} \frac{{\pi_{4} }}{{\pi_{3} }} + h_{24}^{{\text{D,A}}}$$

8 $$Z_{j}^{{\text{A}}} = \pi_{4} \frac{{\left( {h_{31}^{{\text{D,A}}} - h_{33}^{{\text{D,A}}} \frac{{\pi_{1} }}{{\pi_{3} }}} \right)\left[ {\beta^{{\text{D}}} (u^{{\text{D}}} - x_{j}^{{{\text{O}} ,{\text{E}}}} ) - \gamma^{{\text{D}}} (v^{{\text{D}}} - y_{j}^{{{\text{O}} ,{\text{E}}}} )} \right] + \alpha^{{\text{D}}} (v^{{\text{D}}} - y_{j}^{{{\text{O}} ,{\text{E}}}} )\left( {h_{32}^{{\text{D,A}}} - h_{33}^{{\text{D,A}}} \frac{{\pi_{2} }}{{\pi_{3} }}} \right)}}{{\alpha^{{\text{D}}} \beta^{{\text{D}}} \pi_{3} - \pi_{1} \left[ {\beta^{{\text{D}}} (u^{{\text{D}}} - x_{j}^{{{\text{O}} ,{\text{E}}}} ) - \gamma^{{\text{D}}} (v^{{\text{D}}} - y_{j}^{{{\text{O}} ,{\text{E}}}} )} \right] - \pi_{2} \alpha^{{\text{D}}} (v^{{\text{D}}} - y_{j}^{{{\text{O}} ,{\text{E}}}} )}} - h_{33}^{{\text{D,A}}} \frac{{\pi_{4} }}{{\pi_{3} }} + h_{34}^{{\text{D,A}}}.$$

**Figure 2:**
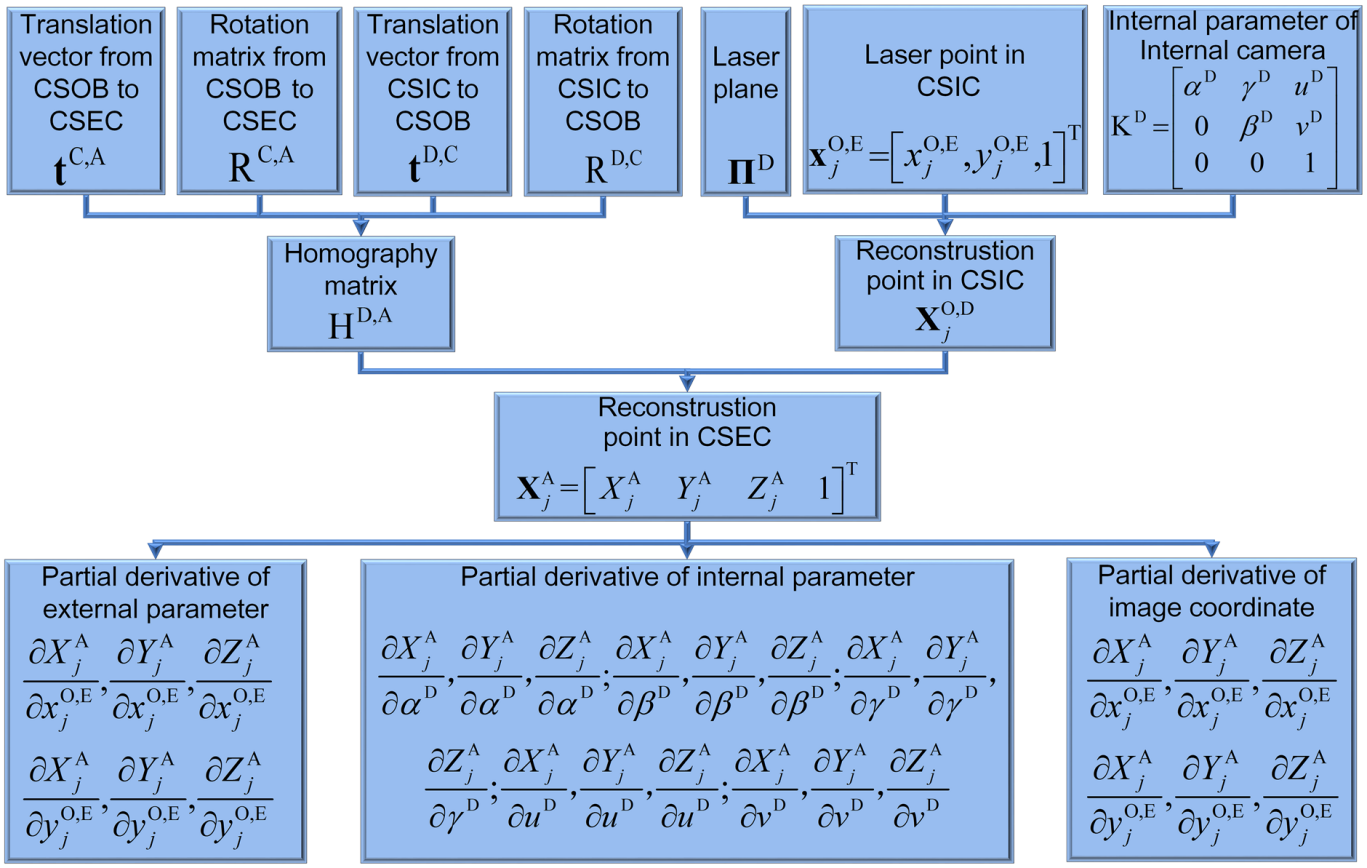
The generations of the partial derivatives of system structure parameters and image coordinates. H^D,A^ is the homography from CSIC to CSEC. ${\mathbf{X}}_{j}^{{{\text{O}},{\text{D}}}}$ is the 3D reconstruction point in CSIC. ${\mathbf{X}}_{j}^{{\text{A}}}$ is the spatial coordinate of the laser point in CSEC. The partial derivatives of the external parameters, internal parameters and image coordinates are generated to the variation ratio of the spatial point in CSEC.

The above process explains the generation process of the reconstruction laser point on the object in CSEC and expresses the elements of the point coordinate by the elements of the extrinsic parameters, intrinsic parameters and image coordinates. Furthermore, we analyze the impacts of the extrinsic parameters, intrinsic parameters and image coordinates on the reconstruction precision by partial derivatives, as the partial derivatives represent the stability of the measurement to the perturbations. Thus, the generations of the partial derivatives of system structure parameters and image coordinates are shown in Fig. 2.

The variation ratio of the spatial point coordinate is evaluated with respect to the extrinsic and intrinsic parameters of the active vision system with the orientation board and two cameras. As the partial derivative represents the variation ratio of function with regard to the argument, the partial derivatives of the extrinsic and intrinsic parameters of the system are generated to indicate the influences of the parameters on the reconstruction precision. Firstly, the relative positions among the external camera, orientation board, and the internal camera are studied by the derivatives. Based on Eqs. (6)–(8), the partial derivatives of the spatial point ${\mathbf{X}}_{j}^{{\text{A}}}$ related to ${\mathbf{t}}^{{\text{C,A}}}$ are.

9 $$\frac{{\partial X_{j}^{{\text{A}}} }}{{\partial {\mathbf{t}}^{{\text{C,A}}} }} = \left[ {\begin{array}{*{20}c} 1 & 0 & 0 \\ \end{array} } \right]^{{\text{T}}}$$

10 $$\frac{{\partial Y_{j}^{{\text{A}}} }}{{\partial {\mathbf{t}}^{{\text{C,A}}} }} = \left[ {\begin{array}{*{20}c} 0 & 1 & 0 \\ \end{array} } \right]^{{\text{T}}}$$

11 $$\frac{{\partial Z_{j}^{{\text{A}}} }}{{\partial {\mathbf{t}}^{{\text{C,A}}} }} = \left[ {\begin{array}{*{20}c} 0 & 0 & 1 \\ \end{array} } \right]^{{\text{T}}}.$$

The partial derivatives of the spatial point ${\mathbf{X}}_{j}^{{\text{A}}}$ related to ${\mathbf{t}}^{{\text{D,C}}}$ are

12 $$\frac{{\partial X_{j}^{{\text{A}}} }}{{\partial {\mathbf{t}}^{{\text{D,C}}} }} = \left[ {\begin{array}{*{20}c} {r_{11}^{{\text{C,A}}} } & {r_{12}^{{\text{C,A}}} } & {r_{13}^{{\text{C,A}}} } \\ \end{array} } \right]^{{\text{T}}}$$

13 $$\frac{{\partial Y_{j}^{{\text{A}}} }}{{\partial {\mathbf{t}}^{{\text{D,C}}} }} = \left[ {\begin{array}{*{20}c} {r_{21}^{{\text{C,A}}} } & {r_{22}^{{\text{C,A}}} } & {r_{23}^{{\text{C,A}}} } \\ \end{array} } \right]^{{\text{T}}}$$

14 $$\frac{{\partial Z_{j}^{{\text{A}}} }}{{\partial {\mathbf{t}}^{{\text{D,C}}} }} = \left[ {\begin{array}{*{20}c} {r_{31}^{{\text{C,A}}} } & {r_{32}^{{\text{C,A}}} } & {r_{33}^{{\text{C,A}}} } \\ \end{array} } \right]^{{\text{T}}}.$$

Then, the influences of the intrinsic parameters of the internal camera on the spatial point ${\mathbf{X}}_{j}^{{\text{A}}}$ are evaluated by partial derivatives. The partial derivatives of the spatial point about $\alpha^{{\text{D}}}$ are

15 $$\frac{{\partial X_{j}^{{\text{A}}} }}{{\partial \alpha^{{\text{D}}} }} = \pi_{4} \frac{{\left[ {\beta^{{\text{D}}} (u^{{\text{D}}} - x_{j}^{{{\text{O}} ,{\text{E}}}} ) - \gamma^{{\text{D}}} (v^{{\text{D}}} - y_{j}^{{{\text{O}} ,{\text{E}}}} )} \right]\left\{ {(v^{{\text{D}}} - y_{j}^{{{\text{O}} ,{\text{E}}}} )\left( {h_{11}^{{\text{D,A}}} \pi_{2} - h_{12}^{{\text{D,A}}} \pi_{1} } \right) - \beta^{{{\text{I}} {\text{I}} {\text{I}} }} \left( {h_{11}^{{\text{D,A}}} \pi_{3} - h_{13}^{{\text{D,A}}} \pi_{1} } \right)} \right\}}}{{\left\{ {\alpha^{{\text{D}}} \beta^{{\text{D}}} \pi_{3} - \pi_{1} \left[ {\beta^{{\text{D}}} (u^{{\text{D}}} - x_{j}^{{{\text{O}} ,{\text{E}}}} ) - \gamma^{{\text{D}}} (v^{{\text{D}}} - y_{j}^{{{\text{O}} ,{\text{E}}}} )} \right] - \pi_{2} \alpha^{{\text{D}}} (v^{{\text{D}}} - y_{j}^{{{\text{O}} ,{\text{E}}}} )} \right\}^{2} }}$$

16 $$\frac{{\partial Y_{j}^{{\text{A}}} }}{{\partial \alpha^{{\text{D}}} }} = \pi_{4} \frac{{\left[ {\beta^{{\text{D}}} (u^{{\text{D}}} - x_{j}^{{{\text{O}} ,{\text{E}}}} ) - \gamma^{{\text{D}}} (v^{{\text{D}}} - y_{j}^{{{\text{O}} ,{\text{E}}}} )} \right]\left\{ {(v^{{\text{D}}} - y_{j}^{{{\text{O}} ,{\text{E}}}} )\left( {h_{21}^{{\text{D,A}}} \pi_{2} - h_{22}^{{\text{D,A}}} \pi_{1} } \right) - \beta^{{{\text{I}} {\text{I}} {\text{I}} }} \left( {h_{21}^{{\text{D,A}}} \pi_{3} - h_{23}^{{\text{D,A}}} \pi_{1} } \right)} \right\}}}{{\left\{ {\alpha^{{\text{D}}} \beta^{{\text{D}}} \pi_{3} - \pi_{1} \left[ {\beta^{{\text{D}}} (u^{{\text{D}}} - x_{j}^{{{\text{O}} ,{\text{E}}}} ) - \gamma^{{\text{D}}} (v^{{\text{D}}} - y_{j}^{{{\text{O}} ,{\text{E}}}} )} \right] - \pi_{2} \alpha^{{\text{D}}} (v^{{\text{D}}} - y_{j}^{{{\text{O}} ,{\text{E}}}} )} \right\}^{2} }}$$

17 $$\frac{{\partial Z_{j}^{{\text{A}}} }}{{\partial \alpha^{{\text{D}}} }} = \pi_{4} \frac{{\left[ {\beta^{{\text{D}}} (u^{{\text{D}}} - x_{j}^{{{\text{O}} ,{\text{E}}}} ) - \gamma^{{\text{D}}} (v^{{\text{D}}} - y_{j}^{{{\text{O}} ,{\text{E}}}} )} \right]\left\{ {(v^{{\text{D}}} - y_{j}^{{{\text{O}} ,{\text{E}}}} )\left( {h_{31}^{{\text{D,A}}} \pi_{2} - h_{32}^{{\text{D,A}}} \pi_{1} } \right) - \beta^{{{\text{I}} {\text{I}} {\text{I}} }} \left( {h_{31}^{{\text{D,A}}} \pi_{3} - h_{33}^{{\text{D,A}}} \pi_{1} } \right)} \right\}}}{{\left\{ {\alpha^{{\text{D}}} \beta^{{\text{D}}} \pi_{3} - \pi_{1} \left[ {\beta^{{\text{D}}} (u^{{\text{D}}} - x_{j}^{{{\text{O}} ,{\text{E}}}} ) - \gamma^{{\text{D}}} (v^{{\text{D}}} - y_{j}^{{{\text{O}} ,{\text{E}}}} )} \right] - \pi_{2} \alpha^{{\text{D}}} (v^{{\text{D}}} - y_{j}^{{{\text{O}} ,{\text{E}}}} )} \right\}^{2} }}.$$

The partial derivatives of the spatial point about $\beta^{{\text{D}}}$ are

18 $$\frac{{\partial X_{j}^{{\text{A}}} }}{{\partial \beta^{{\text{D}}} }} = \pi_{4} \frac{{\alpha^{{\text{D}}} (v^{{\text{D}}} - y_{j}^{{{\text{O}} ,{\text{E}}}} )\left\{ {\gamma^{{\text{D}}} \left( {h_{11}^{{\text{D,A}}} \pi_{3} - h_{13}^{{\text{D,A}}} \pi_{1} } \right) - (u^{{\text{D}}} - x_{j}^{{{\text{O}} ,{\text{E}}}} )\left( {h_{11}^{{\text{D,A}}} \pi_{2} - h_{12}^{{\text{D,A}}} \pi_{1} } \right) - \alpha^{{\text{D}}} \left( {h_{12}^{{\text{D,A}}} \pi_{3} - h_{13}^{{\text{D,A}}} \pi_{2} } \right)} \right\}}}{{\left\{ {\alpha^{{\text{D}}} \beta^{{\text{D}}} \pi_{3} - \pi_{1} \left[ {\beta^{{\text{D}}} (u^{{\text{D}}} - x_{j}^{{{\text{O}} ,{\text{E}}}} ) - \gamma^{{\text{D}}} (v^{{\text{D}}} - y_{j}^{{{\text{O}} ,{\text{E}}}} )} \right] - \pi_{2} \alpha^{{\text{D}}} (v^{{\text{D}}} - y_{j}^{{{\text{O}} ,{\text{E}}}} )} \right\}^{2} }}$$

19 $$\frac{{\partial Y_{j}^{{\text{A}}} }}{{\partial \beta^{{\text{D}}} }} = \pi_{4} \frac{{\alpha^{{\text{D}}} (v^{{\text{D}}} - y_{j}^{{{\text{O}} ,{\text{E}}}} )\left\{ {\gamma^{{\text{D}}} \left( {h_{21}^{{\text{D,A}}} \pi_{3} - h_{23}^{{\text{D,A}}} \pi_{1} } \right) - (u^{{\text{D}}} - x_{j}^{{{\text{O}} ,{\text{E}}}} )\left( {h_{21}^{{\text{D,A}}} \pi_{2} - h_{22}^{{\text{D,A}}} \pi_{1} } \right) - \alpha^{{\text{D}}} \left( {h_{22}^{{\text{D,A}}} \pi_{3} - h_{23}^{{\text{D,A}}} \pi_{2} } \right)} \right\}}}{{\left\{ {\alpha^{{\text{D}}} \beta^{{\text{D}}} \pi_{3} - \pi_{1} \left[ {\beta^{{\text{D}}} (u^{{\text{D}}} - x_{j}^{{{\text{O}} ,{\text{E}}}} ) - \gamma^{{\text{D}}} (v^{{\text{D}}} - y_{j}^{{{\text{O}} ,{\text{E}}}} )} \right] - \pi_{2} \alpha^{{\text{D}}} (v^{{\text{D}}} - y_{j}^{{{\text{O}} ,{\text{E}}}} )} \right\}^{2} }}$$

20 $$\frac{{\partial Z_{j}^{{\text{A}}} }}{{\partial \beta^{{\text{D}}} }} = \pi_{4} \frac{{\alpha^{{\text{D}}} (v^{{\text{D}}} - y_{j}^{{{\text{O}} ,{\text{E}}}} )\left\{ {\gamma^{{\text{D}}} \left( {h_{31}^{{\text{D,A}}} \pi_{3} - h_{33}^{{\text{D,A}}} \pi_{1} } \right) - (u^{{\text{D}}} - x_{j}^{{{\text{O}} ,{\text{E}}}} )\left( {h_{31}^{{\text{D,A}}} \pi_{2} - h_{32}^{{\text{D,A}}} \pi_{1} } \right) - \alpha^{{\text{D}}} \left( {h_{32}^{{\text{D,A}}} \pi_{3} - h_{33}^{{\text{D,A}}} \pi_{2} } \right)} \right\}}}{{\left\{ {\alpha^{{\text{D}}} \beta^{{\text{D}}} \pi_{3} - \pi_{1} \left[ {\beta^{{\text{D}}} (u^{{\text{D}}} - x_{j}^{{{\text{O}} ,{\text{E}}}} ) - \gamma^{{\text{D}}} (v^{{\text{D}}} - y_{j}^{{{\text{O}} ,{\text{E}}}} )} \right] - \pi_{2} \alpha^{{\text{D}}} (v^{{\text{D}}} - y_{j}^{{{\text{O}} ,{\text{E}}}} )} \right\}^{2} }}.$$

The partial derivatives of the spatial point about $\gamma^{{\text{D}}}$ are

21 $$\frac{{\partial X_{j}^{{\text{A}}} }}{{\partial \gamma^{{\text{D}}} }} = \pi_{4} \frac{{\alpha^{{\text{D}}} (v^{{\text{D}}} - y_{j}^{{{\text{O}} ,{\text{E}}}} )\left[ {(v^{{\text{D}}} - y_{j}^{{{\text{O}} ,{\text{E}}}} )\left( {h_{11}^{{\text{D,A}}} \pi_{2} - h_{12}^{{\text{D,A}}} \pi_{1} } \right) - \beta^{{\text{D}}} \left( {h_{11}^{{\text{D,A}}} \pi_{3} - h_{13}^{{\text{D,A}}} \pi_{1} } \right)} \right]}}{{\left\{ {\alpha^{{\text{D}}} \beta^{{\text{D}}} \pi_{3} - \pi_{1} \left[ {\beta^{{\text{D}}} (u^{{\text{D}}} - x_{j}^{{{\text{O}} ,{\text{E}}}} ) - \gamma^{{\text{D}}} (v^{{\text{D}}} - y_{j}^{{{\text{O}} ,{\text{E}}}} )} \right] - \pi_{2} \alpha^{{\text{D}}} (v^{{\text{D}}} - y_{j}^{{{\text{O}} ,{\text{E}}}} )} \right\}^{2} }}$$

22 $$\frac{{\partial Y_{j}^{{\text{A}}} }}{{\partial \gamma^{{\text{D}}} }} = \pi_{4} \frac{{\alpha^{{\text{D}}} (v^{{\text{D}}} - y_{j}^{{{\text{O}} ,{\text{E}}}} )\left[ {(v^{{\text{D}}} - y_{j}^{{{\text{O}} ,{\text{E}}}} )\left( {h_{21}^{{\text{D,A}}} \pi_{2} - h_{22}^{{\text{D,A}}} \pi_{1} } \right) - \beta^{{\text{D}}} \left( {h_{21}^{{\text{D,A}}} \pi_{3} - h_{23}^{{\text{D,A}}} \pi_{1} } \right)} \right]}}{{\left\{ {\alpha^{{\text{D}}} \beta^{{\text{D}}} \pi_{3} - \pi_{1} \left[ {\beta^{{\text{D}}} (u^{{\text{D}}} - x_{j}^{{{\text{O}} ,{\text{E}}}} ) - \gamma^{{\text{D}}} (v^{{\text{D}}} - y_{j}^{{{\text{O}} ,{\text{E}}}} )} \right] - \pi_{2} \alpha^{{\text{D}}} (v^{{\text{D}}} - y_{j}^{{{\text{O}} ,{\text{E}}}} )} \right\}^{2} }}$$

23 $$\frac{{\partial Z_{j}^{{\text{A}}} }}{{\partial \gamma^{{\text{D}}} }} = \pi_{4} \frac{{\alpha^{{\text{D}}} (v^{{\text{D}}} - y_{j}^{{{\text{O}} ,{\text{E}}}} )\left[ {(v^{{\text{D}}} - y_{j}^{{{\text{O}} ,{\text{E}}}} )\left( {h_{31}^{{\text{D,A}}} \pi_{2} - h_{32}^{{\text{D,A}}} \pi_{1} } \right) - \beta^{{\text{D}}} \left( {h_{31}^{{\text{D,A}}} \pi_{3} - h_{33}^{{\text{D,A}}} \pi_{1} } \right)} \right]}}{{\left\{ {\alpha^{{\text{D}}} \beta^{{\text{D}}} \pi_{3} - \pi_{1} \left[ {\beta^{{\text{D}}} (u^{{\text{D}}} - x_{j}^{{{\text{O}} ,{\text{E}}}} ) - \gamma^{{\text{D}}} (v^{{\text{D}}} - y_{j}^{{{\text{O}} ,{\text{E}}}} )} \right] - \pi_{2} \alpha^{{\text{D}}} (v^{{\text{D}}} - y_{j}^{{{\text{O}} ,{\text{E}}}} )} \right\}^{2} }}$$

The partial derivatives of the spatial point about $u^{{\text{D}}}$ are

24 $$\frac{{\partial X_{j}^{{\text{A}}} }}{{\partial u^{{\text{D}}} }} = \pi_{4} \frac{{\alpha^{{\text{D}}} \beta^{{\text{D}}} \left[ {\beta^{{\text{D}}} \left( {h_{11}^{{\text{D,A}}} \pi_{3} - h_{13}^{{\text{D,A}}} \pi_{1} } \right) - (v^{{\text{D}}} - y_{j}^{{{\text{O}} ,{\text{E}}}} )\left( {h_{11}^{{\text{D,A}}} \pi_{2} - h_{12}^{{\text{D,A}}} \pi_{1} } \right)} \right]}}{{\left\{ {\alpha^{{\text{D}}} \beta^{{\text{D}}} \pi_{3} - \pi_{1} \left[ {\beta^{{\text{D}}} (u^{{\text{D}}} - x_{j}^{{{\text{O}} ,{\text{E}}}} ) - \gamma^{{\text{D}}} (v^{{\text{D}}} - y_{j}^{{{\text{O}} ,{\text{E}}}} )} \right] - \pi_{2} \alpha^{{\text{D}}} (v^{{\text{D}}} - y_{j}^{{{\text{O}} ,{\text{E}}}} )} \right\}^{2} }}$$

25 $$\frac{{\partial Y_{j}^{{\text{A}}} }}{{\partial u^{{\text{D}}} }} = \pi_{4} \frac{{\alpha^{{\text{D}}} \beta^{{\text{D}}} \left[ {\beta^{{\text{D}}} \left( {h_{21}^{{\text{D,A}}} \pi_{3} - h_{23}^{{\text{D,A}}} \pi_{1} } \right) - (v^{{\text{D}}} - y_{j}^{{{\text{O}} ,{\text{E}}}} )\left( {h_{21}^{{\text{D,A}}} \pi_{2} - h_{22}^{{\text{D,A}}} \pi_{1} } \right)} \right]}}{{\left\{ {\alpha^{{\text{D}}} \beta^{{\text{D}}} \pi_{3} - \pi_{1} \left[ {\beta^{{\text{D}}} (u^{{\text{D}}} - x_{j}^{{{\text{O}} ,{\text{E}}}} ) - \gamma^{{\text{D}}} (v^{{\text{D}}} - y_{j}^{{{\text{O}} ,{\text{E}}}} )} \right] - \pi_{2} \alpha^{{\text{D}}} (v^{{\text{D}}} - y_{j}^{{{\text{O}} ,{\text{E}}}} )} \right\}^{2} }}$$

26 $$\frac{{\partial Z_{j}^{{\text{A}}} }}{{\partial u^{{\text{D}}} }} = \pi_{4} \frac{{\alpha^{{\text{D}}} \beta^{{\text{D}}} \left[ {\beta^{{\text{D}}} \left( {h_{31}^{{\text{D,A}}} \pi_{3} - h_{33}^{{\text{D,A}}} \pi_{1} } \right) - (v^{{\text{D}}} - y_{j}^{{{\text{O}} ,{\text{E}}}} )\left( {h_{31}^{{\text{D,A}}} \pi_{2} - h_{32}^{{\text{D,A}}} \pi_{1} } \right)} \right]}}{{\left\{ {\alpha^{{\text{D}}} \beta^{{\text{D}}} \pi_{3} - \pi_{1} \left[ {\beta^{{\text{D}}} (u^{{\text{D}}} - x_{j}^{{{\text{O}} ,{\text{E}}}} ) - \gamma^{{\text{D}}} (v^{{\text{D}}} - y_{j}^{{{\text{O}} ,{\text{E}}}} )} \right] - \pi_{2} \alpha^{{\text{D}}} (v^{{\text{D}}} - y_{j}^{{{\text{O}} ,{\text{E}}}} )} \right\}^{2} }}.$$

The partial derivatives of the spatial point about $v^{{\text{D}}}$ are

27 $$\frac{{\partial X_{j}^{{\text{A}}} }}{{\partial v^{{\text{D}}} }} = \pi_{4} \frac{{\alpha^{{\text{D}}} \beta^{{\text{D}}} \left[ {\alpha^{{\text{D}}} \left( {h_{12}^{{\text{D,A}}} \pi_{3} - h_{13}^{{\text{D,A}}} \pi_{2} } \right) - \gamma^{{\text{D}}} \left( {h_{11}^{{\text{D,A}}} \pi_{3} - h_{13}^{{\text{D,A}}} \pi_{1} } \right) + (u^{{\text{D}}} - x_{j}^{{{\text{O}} ,{\text{E}}}} )\left( {h_{11}^{{\text{D,A}}} \pi_{2} - h_{12}^{{\text{D,A}}} \pi_{1} } \right)} \right]}}{{\left\{ {\alpha^{{\text{D}}} \beta^{{\text{D}}} \pi_{3} - \pi_{1} \left[ {\beta^{{\text{D}}} (u^{{\text{D}}} - x_{j}^{{{\text{O}} ,{\text{E}}}} ) - \gamma^{{\text{D}}} (v^{{\text{D}}} - y_{j}^{{{\text{O}} ,{\text{E}}}} )} \right] - \pi_{2} \alpha^{{\text{D}}} (v^{{\text{D}}} - y_{j}^{{{\text{O}} ,{\text{E}}}} )} \right\}^{2} }}$$

28 $$\frac{{\partial Y_{j}^{{\text{A}}} }}{{\partial v^{{\text{D}}} }} = \pi_{4} \frac{{\alpha^{{\text{D}}} \beta^{{\text{D}}} \left[ {\alpha^{{\text{D}}} \left( {h_{22}^{{\text{D,A}}} \pi_{3} - h_{23}^{{\text{D,A}}} \pi_{2} } \right) - \gamma^{{\text{D}}} \left( {h_{21}^{{\text{D,A}}} \pi_{3} - h_{23}^{{\text{D,A}}} \pi_{1} } \right) + (u^{{\text{D}}} - x_{j}^{{{\text{O}} ,{\text{E}}}} )\left( {h_{21}^{{\text{D,A}}} \pi_{2} - h_{22}^{{\text{D,A}}} \pi_{1} } \right)} \right]}}{{\left\{ {\alpha^{{\text{D}}} \beta^{{\text{D}}} \pi_{3} - \pi_{1} \left[ {\beta^{{\text{D}}} (u^{{\text{D}}} - x_{j}^{{{\text{O}} ,{\text{E}}}} ) - \gamma^{{\text{D}}} (v^{{\text{D}}} - y_{j}^{{{\text{O}} ,{\text{E}}}} )} \right] - \pi_{2} \alpha^{{\text{D}}} (v^{{\text{D}}} - y_{j}^{{{\text{O}} ,{\text{E}}}} )} \right\}^{2} }}$$

29 $$\frac{{\partial Z_{j}^{{\text{A}}} }}{{\partial v^{{\text{D}}} }} = \pi_{4} \frac{{\alpha^{{\text{D}}} \beta^{{\text{D}}} \left[ {\alpha^{{\text{D}}} \left( {h_{32}^{{\text{D,A}}} \pi_{3} - h_{33}^{{\text{D,A}}} \pi_{2} } \right) - \gamma^{{\text{D}}} \left( {h_{31}^{{\text{D,A}}} \pi_{3} - h_{33}^{{\text{D,A}}} \pi_{1} } \right) + (u^{{\text{D}}} - x_{j}^{{{\text{O}} ,{\text{E}}}} )\left( {h_{31}^{{\text{D,A}}} \pi_{2} - h_{32}^{{\text{D,A}}} \pi_{1} } \right)} \right]}}{{\left\{ {\alpha^{{\text{D}}} \beta^{{\text{D}}} \pi_{3} - \pi_{1} \left[ {\beta^{{\text{D}}} (u^{{\text{D}}} - x_{j}^{{{\text{O}} ,{\text{E}}}} ) - \gamma^{{\text{D}}} (v^{{\text{D}}} - y_{j}^{{{\text{O}} ,{\text{E}}}} )} \right] - \pi_{2} \alpha^{{\text{D}}} (v^{{\text{D}}} - y_{j}^{{{\text{O}} ,{\text{E}}}} )} \right\}^{2} }}.$$

Finally, as there is noise on the captured image, the influences of the image point ${\mathbf{x}}_{j}^{{\text{O,E}}}$ on the spatial point ${\mathbf{X}}_{j}^{{\text{A}}}$ are also estimated by partial derivatives. The partial derivatives of the spatial point about $x_{j}^{{\text{O,E}}}$ are

30 $$\frac{{\partial X_{j}^{{\text{A}}} }}{{\partial x_{j}^{{{\text{O}} ,{\text{E}}}} }} = \pi_{4} \frac{{\alpha^{{\text{D}}} \beta^{{\text{D}}} \left[ {(v^{{\text{D}}} - y_{j}^{{{\text{O}} ,{\text{E}}}} )\left( {h_{11}^{{\text{D,A}}} \pi_{2} - h_{12}^{{\text{D,A}}} \pi_{1} } \right) - \beta^{{\text{D}}} \left( {h_{11}^{{\text{D,A}}} \pi_{3} - h_{13}^{{\text{D,A}}} \pi_{1} } \right)} \right]}}{{\left\{ {\alpha^{{\text{D}}} \beta^{{\text{D}}} \pi_{3} - \pi_{1} \left[ {\beta^{{\text{D}}} (u^{{\text{D}}} - x_{j}^{{{\text{O}} ,{\text{E}}}} ) - \gamma^{{\text{D}}} (v^{{\text{D}}} - y_{j}^{{{\text{O}} ,{\text{E}}}} )} \right] - \pi_{2} \alpha^{{\text{D}}} (v^{{\text{D}}} - y_{j}^{{{\text{O}} ,{\text{E}}}} )} \right\}^{2} }}$$

31 $$\frac{{\partial Y_{j}^{{\text{A}}} }}{{\partial x_{j}^{{{\text{O}} ,{\text{E}}}} }} = \pi_{4} \frac{{\alpha^{{\text{D}}} \beta^{{\text{D}}} \left[ {(v^{{\text{D}}} - y_{j}^{{{\text{O}} ,{\text{E}}}} )\left( {h_{21}^{{\text{D,A}}} \pi_{2} - h_{22}^{{\text{D,A}}} \pi_{1} } \right) - \beta^{{\text{D}}} \left( {h_{21}^{{\text{D,A}}} \pi_{3} - h_{23}^{{\text{D,A}}} \pi_{1} } \right)} \right]}}{{\left\{ {\alpha^{{\text{D}}} \beta^{{\text{D}}} \pi_{3} - \pi_{1} \left[ {\beta^{{\text{D}}} (u^{{\text{D}}} - x_{j}^{{{\text{O}} ,{\text{E}}}} ) - \gamma^{{\text{D}}} (v^{{\text{D}}} - y_{j}^{{{\text{O}} ,{\text{E}}}} )} \right] - \pi_{2} \alpha^{{\text{D}}} (v^{{\text{D}}} - y_{j}^{{{\text{O}} ,{\text{E}}}} )} \right\}^{2} }}$$

32 $$\frac{{\partial Z_{j}^{{\text{A}}} }}{{\partial x_{j}^{{{\text{O}} ,{\text{E}}}} }} = \pi_{4} \frac{{\alpha^{{\text{D}}} \beta^{{\text{D}}} \left[ {(v^{{\text{D}}} - y_{j}^{{{\text{O}} ,{\text{E}}}} )\left( {h_{31}^{{\text{D,A}}} \pi_{2} - h_{32}^{{\text{D,A}}} \pi_{1} } \right) - \beta^{{\text{D}}} \left( {h_{31}^{{\text{D,A}}} \pi_{3} - h_{33}^{{\text{D,A}}} \pi_{1} } \right)} \right]}}{{\left\{ {\alpha^{{\text{D}}} \beta^{{\text{D}}} \pi_{3} - \pi_{1} \left[ {\beta^{{\text{D}}} (u^{{\text{D}}} - x_{j}^{{{\text{O}} ,{\text{E}}}} ) - \gamma^{{\text{D}}} (v^{{\text{D}}} - y_{j}^{{{\text{O}} ,{\text{E}}}} )} \right] - \pi_{2} \alpha^{{\text{D}}} (v^{{\text{D}}} - y_{j}^{{{\text{O}} ,{\text{E}}}} )} \right\}^{2} }}$$

The partial derivatives of the spatial point about $y_{j}^{{\text{O,E}}}$ are

33 $$\frac{{\partial X_{j}^{{\text{A}}} }}{{\partial y_{j}^{{{\text{O}} ,{\text{E}}}} }} = \pi_{4} \frac{{\alpha^{{\text{D}}} \beta^{{\text{D}}} \left[ {\gamma^{{\text{D}}} \left( {h_{11}^{{\text{D,A}}} \pi_{3} - h_{13}^{{\text{D,A}}} \pi_{1} } \right) - \alpha^{{\text{D}}} \left( {h_{12}^{{\text{D,A}}} \pi_{3} - h_{13}^{{\text{D,A}}} \pi_{2} } \right) - (u^{{\text{D}}} - x_{j}^{{{\text{O}} ,{\text{E}}}} )\left( {h_{11}^{{\text{D,A}}} \pi_{2} - h_{12}^{{\text{D,A}}} \pi_{1} } \right)} \right]}}{{\left\{ {\alpha^{{\text{D}}} \beta^{{\text{D}}} \pi_{3} - \pi_{1} \left[ {\beta^{{\text{D}}} (u^{{\text{D}}} - x_{j}^{{{\text{O}} ,{\text{E}}}} ) - \gamma^{{\text{D}}} (v^{{\text{D}}} - y_{j}^{{{\text{O}} ,{\text{E}}}} )} \right] - \pi_{2} \alpha^{{\text{D}}} (v^{{\text{D}}} - y_{j}^{{{\text{O}} ,{\text{E}}}} )} \right\}^{2} }}$$

34 $$\frac{{\partial Y_{j}^{{\text{A}}} }}{{\partial y_{j}^{{{\text{O}} ,{\text{E}}}} }} = \pi_{4} \frac{{\alpha^{{\text{D}}} \beta^{{\text{D}}} \left[ {\gamma^{{\text{D}}} \left( {h_{21}^{{\text{D,A}}} \pi_{3} - h_{23}^{{\text{D,A}}} \pi_{1} } \right) - \alpha^{{\text{D}}} \left( {h_{22}^{{\text{D,A}}} \pi_{3} - h_{23}^{{\text{D,A}}} \pi_{2} } \right) - (u^{{\text{D}}} - x_{j}^{{{\text{O}} ,{\text{E}}}} )\left( {h_{21}^{{\text{D,A}}} \pi_{2} - h_{22}^{{\text{D,A}}} \pi_{1} } \right)} \right]}}{{\left\{ {\alpha^{{\text{D}}} \beta^{{\text{D}}} \pi_{3} - \pi_{1} \left[ {\beta^{{\text{D}}} (u^{{\text{D}}} - x_{j}^{{{\text{O}} ,{\text{E}}}} ) - \gamma^{{\text{D}}} (v^{{\text{D}}} - y_{j}^{{{\text{O}} ,{\text{E}}}} )} \right] - \pi_{2} \alpha^{{\text{D}}} (v^{{\text{D}}} - y_{j}^{{{\text{O}} ,{\text{E}}}} )} \right\}^{2} }}$$

35 $$\frac{{\partial Z_{j}^{{\text{A}}} }}{{\partial y_{j}^{{{\text{O}} ,{\text{E}}}} }} = \pi_{4} \frac{{\alpha^{{\text{D}}} \beta^{{\text{D}}} \left[ {\gamma^{{\text{D}}} \left( {h_{31}^{{\text{D,A}}} \pi_{3} - h_{13}^{{\text{D,A}}} \pi_{1} } \right) - \alpha^{{\text{D}}} \left( {h_{32}^{{\text{D,A}}} \pi_{3} - h_{33}^{{\text{D,A}}} \pi_{2} } \right) - (u^{{\text{D}}} - x_{j}^{{{\text{O}} ,{\text{E}}}} )\left( {h_{31}^{{\text{D,A}}} \pi_{2} - h_{32}^{{\text{D,A}}} \pi_{1} } \right)} \right]}}{{\left\{ {\alpha^{{\text{D}}} \beta^{{\text{D}}} \pi_{3} - \pi_{1} \left[ {\beta^{{\text{D}}} (u^{{\text{D}}} - x_{j}^{{{\text{O}} ,{\text{E}}}} ) - \gamma^{{\text{D}}} (v^{{\text{D}}} - y_{j}^{{{\text{O}} ,{\text{E}}}} )} \right] - \pi_{2} \alpha^{{\text{D}}} (v^{{\text{D}}} - y_{j}^{{{\text{O}} ,{\text{E}}}} )} \right\}^{2} }}.$$

The influence factors including the translation vectors, the intrinsic parameters of the internal camera, and the related image projection point are considered in the analysis model. The partial derivatives of the above arguments are generated to further conduct the results of the precision analysis and experimentation.

## Results of precision analysis and experimentation

The structural parameters of the system and image coordinates contribute to the positioning accuracy of the reconstruction with the 3D orientation board and two cameras. The experiments are performed by a camera with the resolution of 1280 × 960, the focal length of 8 mm, the horizontal view of 41.7°, the vertical view of 31.8°, and the sub-pixel-level accuracy of laser projection. Therefore, the impacts of 3 main factors of the system are analyzed precisely, including the translation vectors from CSEC to CSOB and from CSOB to CSIC, intrinsic parameters of the internal camera and the image coordinate of the feature point.

### Translation vector

The extrinsic parameters obtained in the calibration process of the active vision system are demonstrated in Table 1. According to Eqs. (12)–(14), for the increasing translation vector from CSOB to CSIC, the partial derivatives are the elements in the rotation matrix of the coordinate transformation from the CSEC to CSOB. As the rotation matrix is a unit orthogonal matrix, that is, the absolute value of each element is not greater than 1. As a result of Eqs. (9)–(11), the coordinates of the reconstruction feature points are linearly related to the CSOB-CSIC translation vector.

**Table 1:** Extrinsic parameters of cameras. R^D,C^ and **t**^D,C^ are the rotation matrix and translation vector from CSIC to CSOB. R^C,A^ and **t**^C,A^ are the rotation matrix and translation vector from CSOB to CSEC.

Variable	R^D,C^	t^D,C^	R^C,A^	t^C,A^
Parameter	$$\left[ {\begin{array}{*{20}l} { - 0.9981} \hfill & {0.0623} \hfill & {0.0037} \hfill \\ {0.0035} \hfill & { - 0.0037} \hfill & {0.9999} \hfill \\ { - 0.0621} \hfill & { - 0.9981} \hfill & { - 0.0035} \hfill \\ \end{array} } \right]$$	$$\left[ {\begin{array}{*{20}l} {222.49} \hfill & {415.47} \hfill & {131.35} \hfill \\ \end{array} } \right]^{{\text{T}}}$$	$$\left[ {\begin{array}{*{20}l} { - 0.8034} \hfill & {0.5953} \hfill & { - 0.0124} \hfill \\ { - 0.1248} \hfill & { - 0.1887} \hfill & { - 0.9741} \hfill \\ { - 0.5822} \hfill & {0.7811} \hfill & { - 0.2257} \hfill \\ \end{array} } \right]$$	$$\left[ {\begin{array}{*{20}l} { - 38.40} \hfill & {369.83} \hfill & {604.14} \hfill \\ \end{array} } \right]^{{\text{T}}}$$

**Figure 3:**
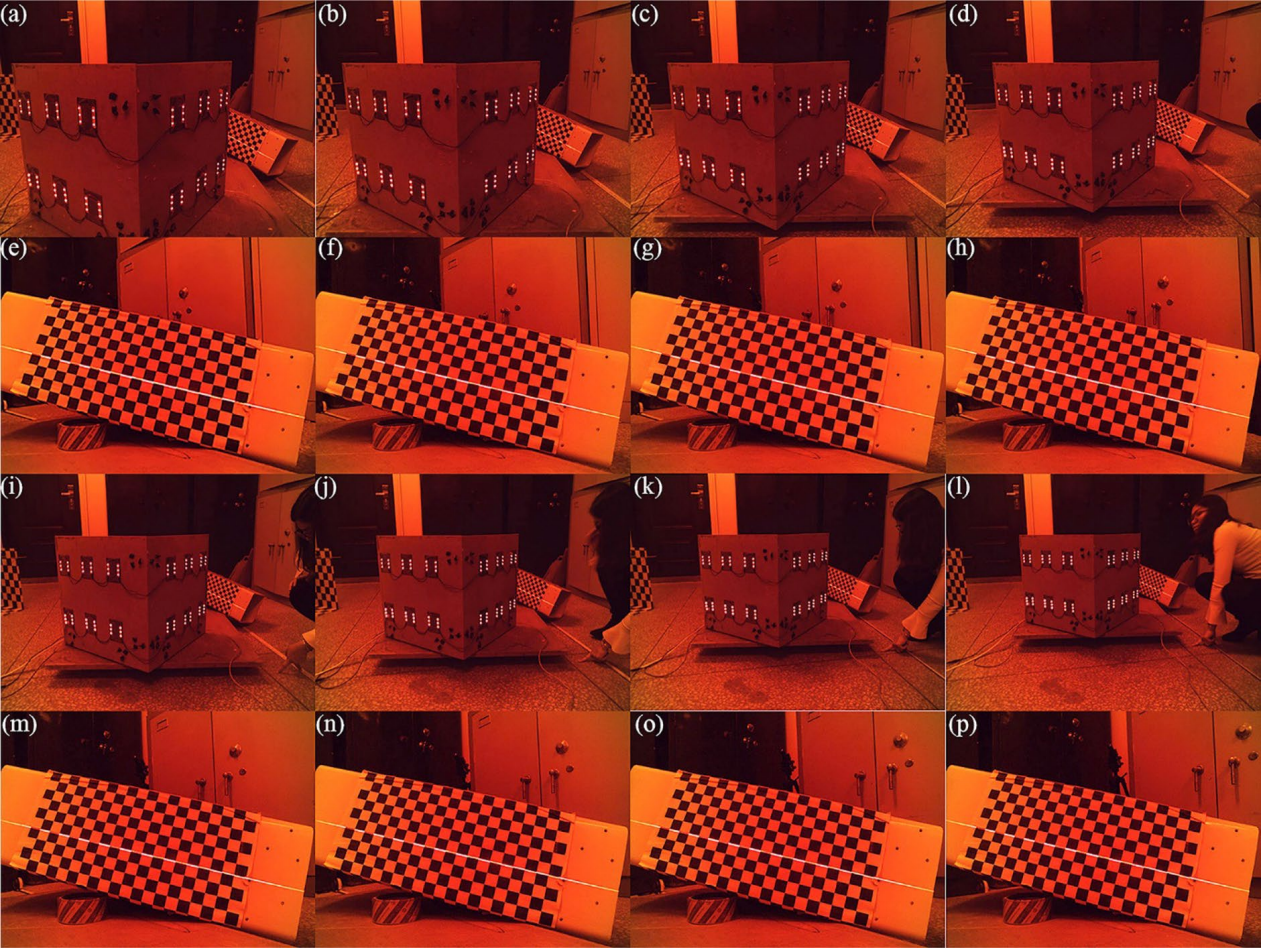
Experimental images of the internal and the external cameras. (**a**–**d**) the experimental images of the external camera. The camera-board distances are 600, 700, 800, 900 mm. (**i**–**l**) the experimental images of the external camera. The camera-board distances are 1000, 1100, 1200, 1300 mm. (**e**–**h**) and (**m**–**p**) the experimental images of the internal camera related to (**a**–**d**) and (**i**–**l**).

**Figure 4:**
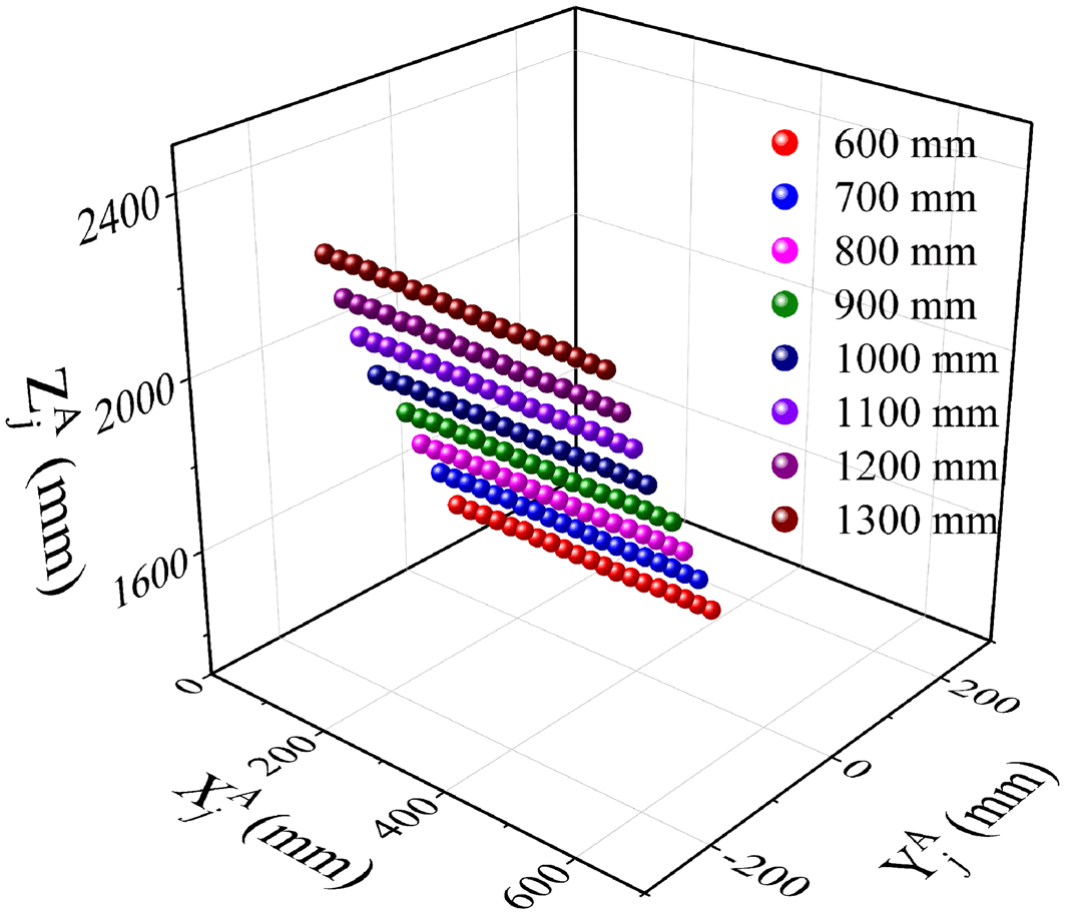
The reconstruction coordinates of the laser points on the chessboard pattern. The distance between the external camera and the cubic board is 600–1300 mm with 100 mm interval. The spatial coordinates of reconstruction points increase evenly with the growth of the distance from CSOB to CSEC.

In addition, a series of verification experiments are performed to analyze the relationship between the spatial coordinate of reconstruction feature point and the CSEC to CSOB translation vectors. Firstly, the distance between the 3D orientation board and the measured object is controlled to be a constant. In this way, the image coordinate of the projection point does not vary in the test. Then, the experiments are carried out for the different distances between the CSOB and CSEC. The experimental process is described in Fig. 3. Figure 3a–d and i–l are the images captured by the external camera, and the distance between the CSOB and CSEC are 600 to 900 mm and 1000 to 1300 mm with the 100 mm interval respectively. Figure 3e–h and m–p are obtained by the internal camera, and the object is located on the same position. The reconstruction coordinates of the laser projections on the chessboard pattern are shown in Fig. 4. It demonstrates that the spatial coordinates of reconstruction points increase evenly with the growth of the distance from CSOB to CSEC. The experiment results prove the conclusion that the reconstruction coordinates of the laser points linearly increase with the rising distance between the external camera and the orientation board, which is consistent with the result of the partial derivatives of translation vectors.

### Intrinsic parameter

The simulation analysis of the intrinsic parameters is based on the data obtained from the calibration experiment. The calibration experiment is performed when the external camera is 600 mm away from the 3D orientation board. The intrinsic parameters are listed in Table 2. According to the data in the Table 2, the scopes of parameters in the simulation analysis are selected reasonably.

**Table 2:** Intrinsic parameters of the internal camera. *α*^D^, *β*^D^ are the pixel-dimension focal lengths of the internal camera in *x* and *y* direction. *γ*^D^ is the skew parameter. *u*^D^, *v*^D^ are the pixel-dimension coordinates of the principal point.

Parameters	*α*^D^	*β*^D^	*u*^D^	*v*^D^	*γ*^D^
Internal camera	1172.91	1165.96	664.54	550.49	0.50

**Figure 5:**
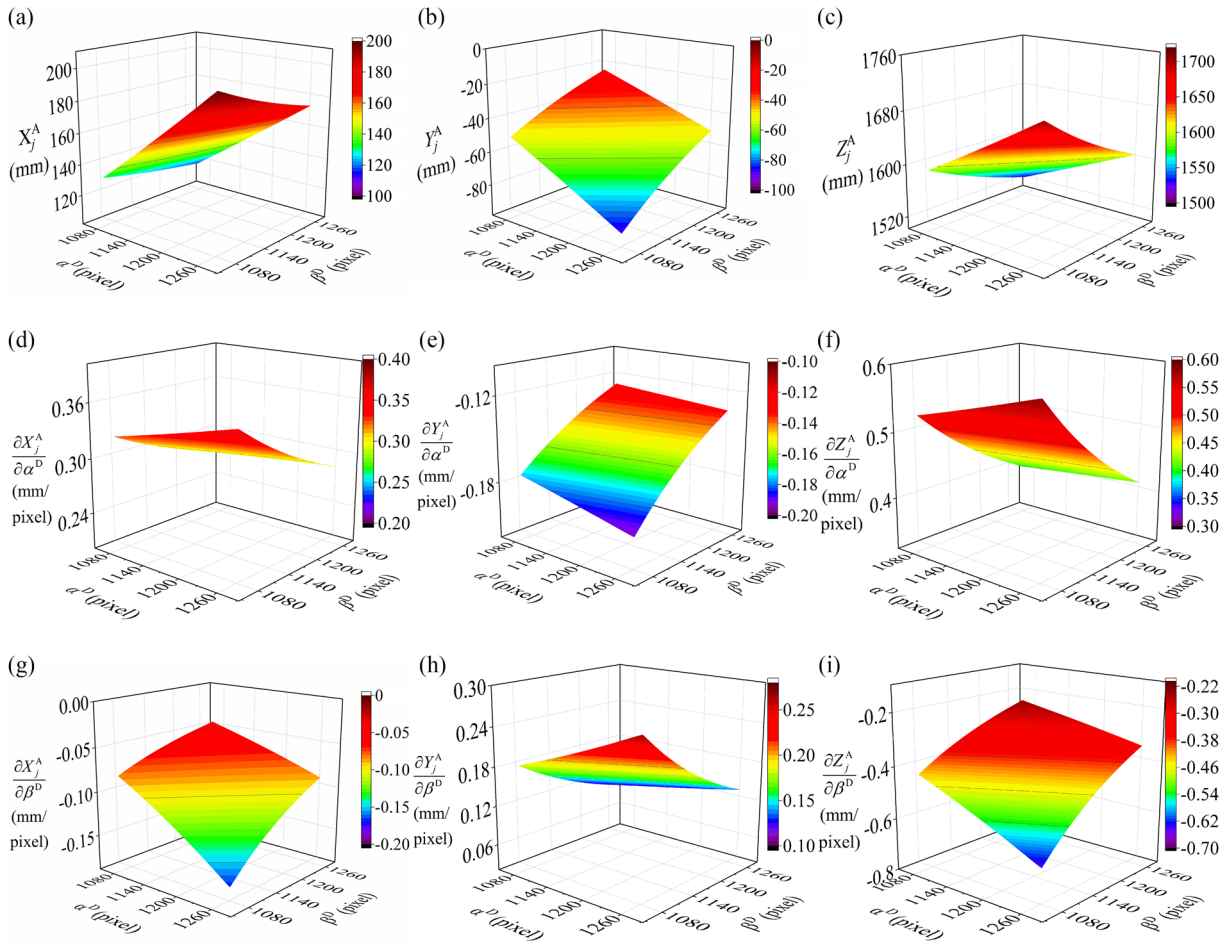
Simulation results of the intrinsic parameters $\alpha^{{\text{D}}}$, $\beta^{{\text{D}}}$. (**a**–**c**) the relationship between the coordinates $X_{j}^{{\text{A}}}$, $Y_{j}^{{\text{A}}}$, $Z_{j}^{{\text{A}}}$ and the intrinsic parameters $\alpha^{{\text{D}}}$, $\beta^{{\text{D}}}$. (**d**–**f**) the relationship between the partial derivatives $\partial X_{j}^{{\text{A}}} {/}\partial \alpha^{{\text{D}}}$, $\partial Y_{j}^{{\text{A}}} {/}\partial \alpha^{{\text{D}}}$, $\partial Z_{j}^{{\text{A}}} {/}\partial \alpha^{{\text{D}}}$ and the intrinsic parameters $\alpha^{{\text{D}}}$, $\beta^{{\text{D}}}$. (**g**–**i**) the relationship between the partial derivatives $\partial X_{j}^{{\text{A}}} {/}\partial \beta^{{\text{D}}}$, $\partial Y_{j}^{{\text{A}}} {/}\partial \beta^{{\text{D}}}$, $\partial Z_{j}^{{\text{A}}} {/}\partial \beta^{{\text{D}}}$ and the intrinsic parameters $\alpha^{{\text{D}}}$, $\beta^{{\text{D}}}$.

**Figure 6:**
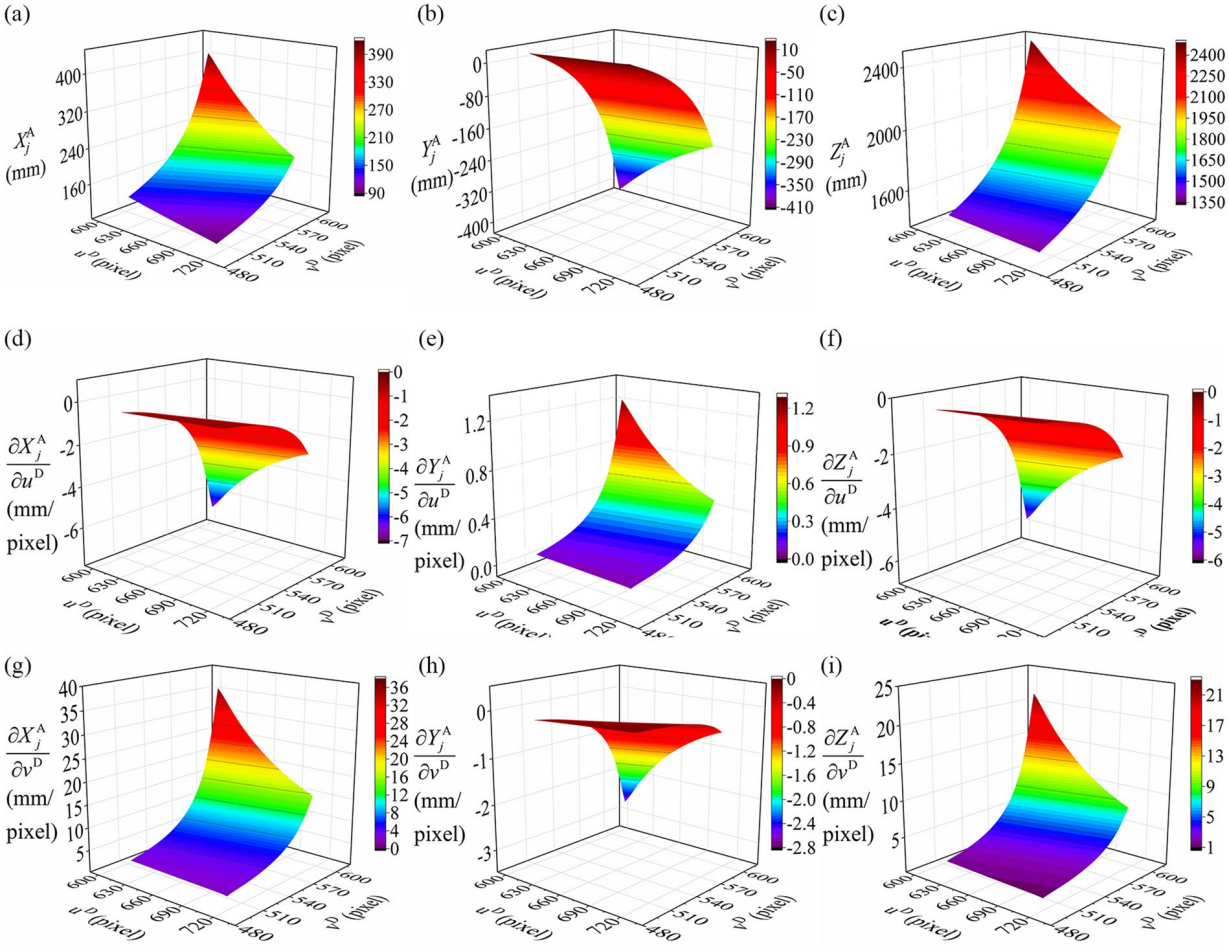
Simulation results of the intrinsic parameters $u^{{\text{D}}}$, $v^{{\text{D}}}$. (**a**–**c**) the relationship between the coordinates $X_{j}^{{\text{A}}}$, $Y_{j}^{{\text{A}}}$, $Z_{j}^{{\text{A}}}$ and the intrinsic parameters $u^{{\text{D}}}$, $v^{{\text{D}}}$. (**d**–**f**) the relationship between the partial derivatives $\partial X_{j}^{{\text{A}}} {/}\partial u^{{\text{D}}}$, $\partial Y_{j}^{{\text{A}}} {/}\partial u^{{\text{D}}}$, $\partial Z_{j}^{{\text{A}}} {/}\partial u^{{\text{D}}}$ and the intrinsic parameters $u^{{\text{D}}}$, $v^{{\text{D}}}$. (**g**–**i**) the relationship between the partial derivatives $\partial X_{j}^{{\text{A}}} {/}\partial v^{{\text{D}}}$, $\partial Y_{j}^{{\text{A}}} {/}\partial v^{{\text{D}}}$, $\partial Z_{j}^{{\text{A}}} {/}\partial v^{{\text{D}}}$ and the intrinsic parameters $u^{{\text{D}}}$, $v^{{\text{D}}}$.

**Figure 7:**
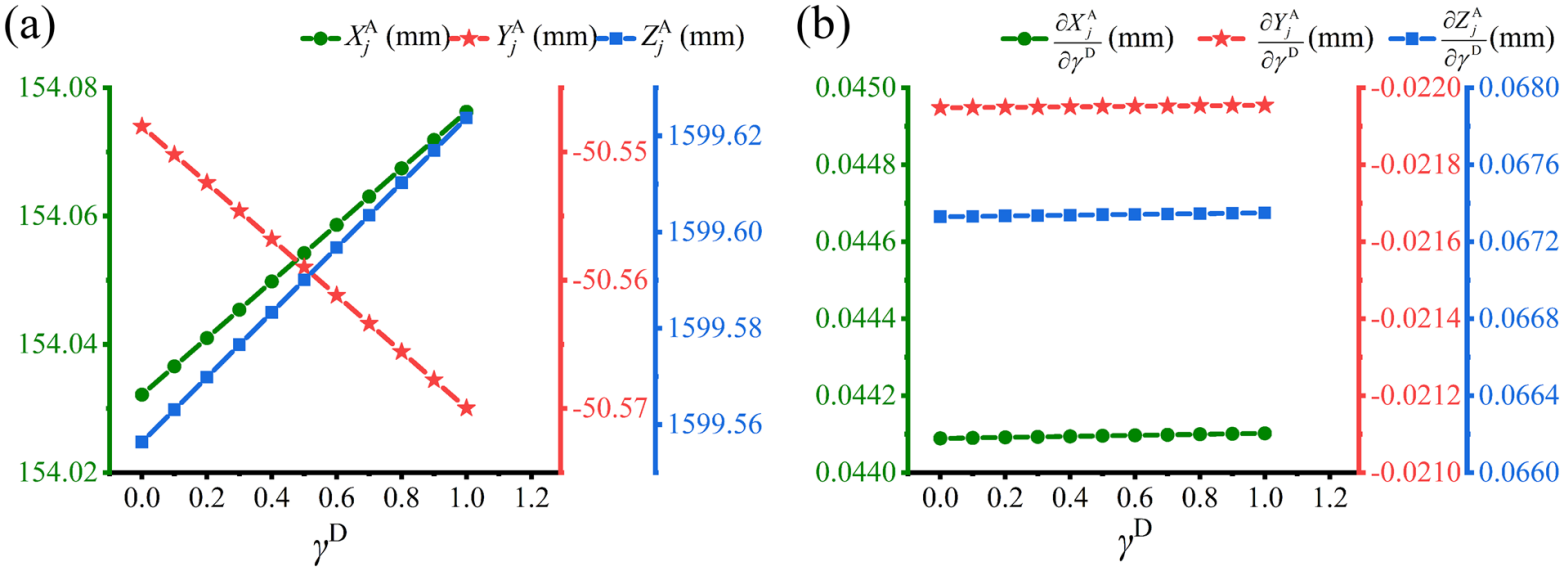
Simulation results of the intrinsic parameters $\gamma^{{\text{D}}}$. (**a**) The relationship between the coordinates $X_{j}^{{\text{A}}}$, $Y_{j}^{{\text{A}}}$, $Z_{j}^{{\text{A}}}$ and the intrinsic parameter $\gamma^{{\text{D}}}$. (**b**) The relationship between the partial derivatives $\partial X_{j}^{{\text{A}}} {/}\partial \gamma^{{\text{D}}}$, $\partial Y_{j}^{{\text{A}}} {/}\partial \gamma^{{\text{D}}}$, $\partial Z_{j}^{{\text{A}}} {/}\partial \gamma^{{\text{D}}}$ and the intrinsic parameter $\gamma^{{\text{D}}}$.

The simulation results of intrinsic parameters are shown in Figs. 5, 6 and 7. Figure 5a–c illustrate the three coordinates of the reconstruction feature points varying with $\alpha^{{\text{D}}}$ and $\beta^{{\text{D}}}$. In Fig. 5a–c, there are several observed tendencies. For the increasing value of $\alpha^{{\text{D}}}$, $X_{j}^{{\text{A}}}$ value of the reconstructed feature point increases steadily. $Y_{j}^{{\text{A}}}$ falls gradually, and $Z_{j}^{{\text{A}}}$ value rises slightly. It is worth noting that the value of $Y_{j}^{{\text{A}}}$ is negative and its absolute value is also increasing. However, the variation trends of $X_{j}^{{\text{A}}}$, $Y_{j}^{{\text{A}}}$, $Z_{j}^{{\text{A}}}$ values are the opposite to the above ones, with the increment of $\beta^{{\text{D}}}$. In order to have a deep insight into the effects of $\alpha^{{\text{D}}}$ and $\beta^{{\text{D}}}$, Fig. 5d–i further discusses the trends of the variations of partial derivatives. It aims to describe the speed of parameter variation by the partial derivative. When the value of $\alpha^{{\text{D}}}$ goes up in Fig. 5d–f, the partial derivative $\partial X_{j}^{{\text{A}}} {/}\partial \alpha^{{\text{D}}}$ is positive, and also shows an increasing trend. However, the partial derivative $\partial Y_{j}^{{\text{A}}} {/}\partial \alpha^{{\text{D}}}$ is negative and declines slightly. The partial derivative $\partial Z_{j}^{{\text{A}}} {/}\partial \alpha^{{\text{D}}}$ is positive and grows up gradually.

For the rising value of $\beta^{{\text{D}}}$, the partial derivative $\partial X_{j}^{{\text{A}}} {/}\partial \alpha^{{\text{D}}}$ declines gradually. The partial derivative $\partial Y_{j}^{{\text{A}}} {/}\partial \alpha^{{\text{D}}}$ rises slightly and the partial derivative $\partial Z_{j}^{{\text{A}}} {/}\partial \alpha^{{\text{D}}}$ decreases slightly. The variation trend in Fig. 5d–f is correspondingly opposite to that in Fig. 5g–i. Nevertheless, it’s remarkable that each partial derivative tends to be zero when the value of $\alpha^{{\text{D}}}$ decreases and $\beta^{{\text{D}}}$ increases.

Figure 6 describes that the influences of $u^{{\text{D}}}$ and $v^{{\text{D}}}$ on the spatial coordinates of the reconstruction feature point and the partial derivatives, respectively. In Fig. 6a–c, for the increasing $u^{{\text{D}}}$, there is a downward trend of $X_{j}^{{\text{A}}}$ and $Z_{j}^{{\text{A}}}$ of the reconstruction feature point. The $Y_{j}^{{\text{A}}}$ value raises gradually. However, the variation trend of $X_{j}^{{\text{A}}}$, $Y_{j}^{{\text{A}}}$ and $Z_{j}^{{\text{A}}}$ values are opposite for the increasing $v^{{\text{D}}}$. When $u^{{\text{D}}}$ rises in Fig. 6d–f, the partial derivatives $\partial X_{j}^{{\text{A}}} {/}\partial u^{{\text{D}}}$, $\partial Z_{j}^{{\text{A}}} {/}\partial u^{{\text{D}}}$ increase steadily. But the partial derivative $\partial Y_{j}^{{\text{A}}} {/}\partial u^{{\text{D}}}$ decreases slightly. Furthermore, when $v^{{\text{D}}}$ increases, the partial derivatives $\partial X_{j}^{{\text{A}}} {/}\partial v^{{\text{D}}}$,$\partial Z_{j}^{{\text{A}}} {/}\partial v^{{\text{D}}}$ decrease steadily and the partial derivative $\partial Y_{j}^{{\text{A}}} {/}\partial v^{{\text{D}}}$ rises slightly. The trends of partial derivatives in Fig. 6g–i provide contrary variations to the ones in Fig. 6d–f.

Finally, Fig. 7 describes the variation trends of the spatial coordinates and the partial derivatives about the twist parameter $\gamma^{{\text{D}}}$. In Fig. 7a, the three plotlines show that the spatial coordinates are linearly related to the twist parameter $\gamma^{{\text{D}}}$. The coordinates of $X_{j}^{{\text{A}}}$ and $Z_{j}^{{\text{A}}}$ increase and $Y_{j}^{{\text{A}}}$ derivative shown in Fig. 7b. The partial derivatives limited within a narrow range for the 0–1 scope of $\gamma^{{\text{D}}}$. The above results are in accord with the trends of partial $\partial X_{j}^{{\text{A}}} {/}\partial \gamma^{{\text{D}}}$ and $\partial Z_{j}^{{\text{A}}} {/}\partial \gamma^{{\text{D}}}$ are positive, whereas $\partial Y_{j}^{{\text{A}}} {/}\partial \gamma^{{\text{D}}}$ is negative. All the partial derivatives are around 0.

### Image point

**Figure 8:**
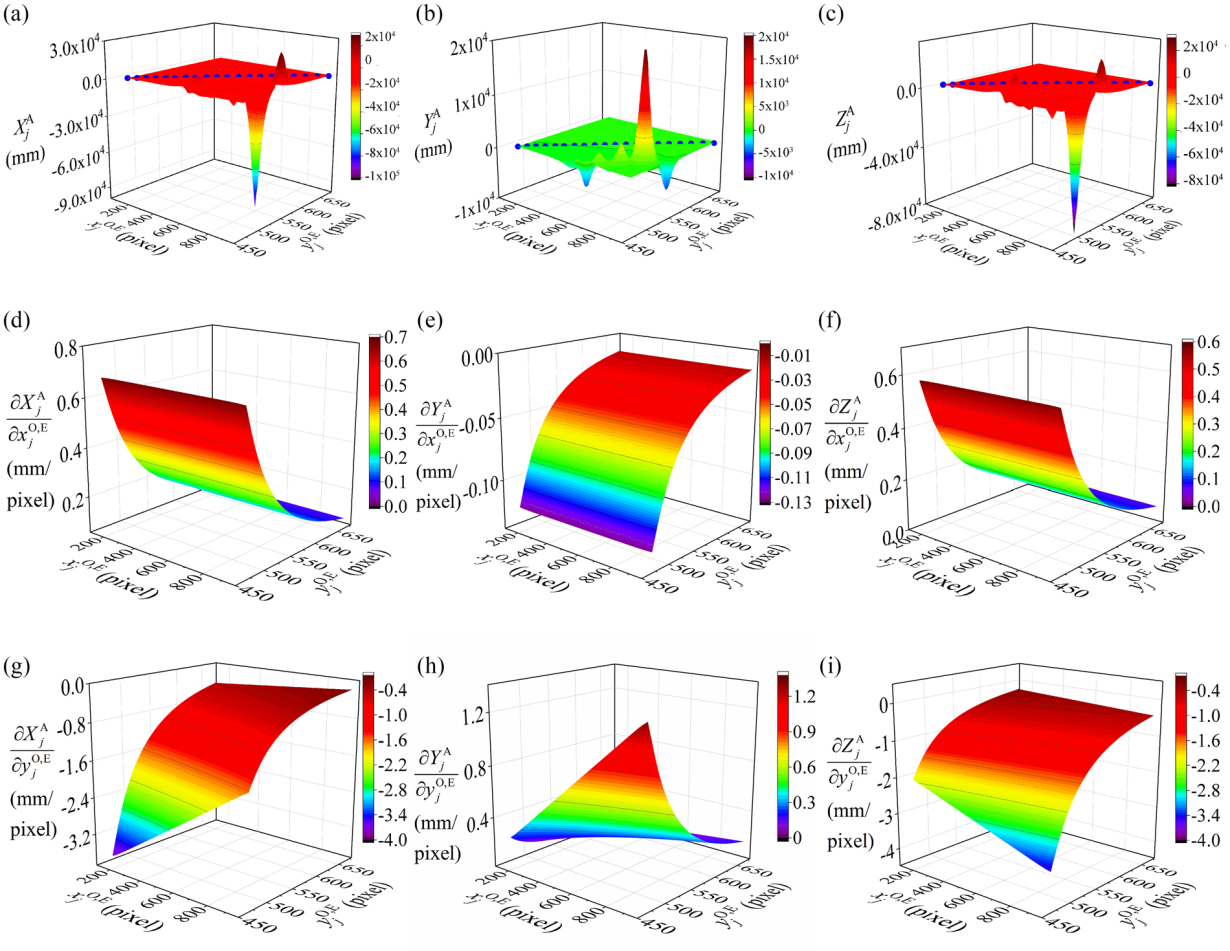
Verification experiments and simulation results of the coordinates of the image point. (**a**–**c**) the relationship between the coordinates $X_{j}^{{\text{A}}}$, $Y_{j}^{{\text{A}}}$, $Z_{j}^{{\text{A}}}$ and the image coordinates $x_{j}^{{{\text{O}} ,{\text{E}}}}$, $y_{j}^{{{\text{O}} ,{\text{E}}}}$. (**d**–**f**) the relationship between the partial derivatives $\partial X_{j}^{{\text{A}}} {/}\partial x_{j}^{{{\text{O}} ,{\text{E}}}}$, $\partial Y_{j}^{{\text{A}}} {/}\partial x_{j}^{{{\text{O}} ,{\text{E}}}}$, $\partial Z_{j}^{{\text{A}}} {/}\partial x_{j}^{{{\text{O}} ,{\text{E}}}}$ and the image coordinates $x_{j}^{{{\text{O}} ,{\text{E}}}}$, $y_{j}^{{{\text{O}} ,{\text{E}}}}$. (**g**–**i**) the relationship between the partial derivatives $\partial X_{j}^{{\text{A}}} {/}\partial x_{j}^{{{\text{O}} ,{\text{E}}}}$, $\partial Y_{j}^{{\text{A}}} {/}\partial x_{j}^{{{\text{O}} ,{\text{E}}}}$, $\partial Z_{j}^{{\text{A}}} {/}\partial x_{j}^{{{\text{O}} ,{\text{E}}}}$ and the image coordinates $x_{j}^{{{\text{O}} ,{\text{E}}}}$, $y_{j}^{{{\text{O}} ,{\text{E}}}}$.

In order to investigate the influences of the image coordinates on the spatial coordinates of reconstruction points, the simulation analysis and verification experiment are carried out and the results are shown in Fig. 8. Figure 8a–c present the great influence of image coordinates on the spatial coordinates of reconstruction points. There is evidently rising or falling phenomenon. Moreover, the experiments are implemented to test the simulation model. In the experiment, the image coordinates of the laser projections that locate on the same laser plane are adopted to the reconstruction. It can be found that the blue dots in Fig. 8a–c are all in the surface obtained by the simulation analysis. So the conclusion can be drawn that the simulation analysis results are consistent with the experiments.

The relationships between the image coordinates and the partial derivatives are illustrated in Fig. 8d–i. $\partial X_{j}^{{\text{A}}} {/}\partial x_{j}^{{{\text{O}} ,{\text{E}}}}$ and $\partial Z_{j}^{{\text{A}}} {/}\partial x_{j}^{{{\text{O}} ,{\text{E}}}}$ show the augmentation and $\partial Y_{j}^{{\text{A}}} {/}\partial x_{j}^{{{\text{O}} ,{\text{E}}}}$ descends slightly with the increment of $x_{j}^{{{\text{O}} ,{\text{E}}}}$. However, the values of $\partial X_{j}^{{\text{A}}} {/}\partial y_{j}^{{{\text{O}} ,{\text{E}}}}$ and $\partial Y_{j}^{{\text{A}}} {/}\partial y_{j}^{{{\text{O}} ,{\text{E}}}}$ grow up and $\partial Z_{j}^{{\text{A}}} {/}\partial y_{j}^{{{\text{O}} ,{\text{E}}}}$ decreases significantly. When $y_{j}^{{{\text{O}} ,{\text{E}}}}$ increases, $\partial X_{j}^{{\text{A}}} {/}\partial x_{j}^{{{\text{O}} ,{\text{E}}}}$ and $\partial Y_{j}^{{\text{A}}} {/}\partial x_{j}^{{{\text{O}} ,{\text{E}}}}$ descend and $\partial Z_{j}^{{\text{A}}} {/}\partial x_{j}^{{{\text{O}} ,{\text{E}}}}$ ascends obviously, Nevertheless, the values of $\partial X_{j}^{{\text{A}}} {/}\partial y_{j}^{{{\text{O}} ,{\text{E}}}}$ and $\partial Z_{j}^{{\text{A}}} {/}\partial y_{j}^{{{\text{O}} ,{\text{E}}}}$ rise and $\partial Y_{j}^{{\text{A}}} {/}\partial y_{j}^{{{\text{O}} ,{\text{E}}}}$ falls down gradually. Generally, the six partial derivatives tend to zero with the increasing value of $y_{j}^{{{\text{O}} ,{\text{E}}}}$.

For the situation of adding one extra camera, the chained-form system should give one cubic board for the original external camera. Then, the original external camera is considered as the new internal camera inside the cubic board in the new system. The added camera is considered as the new external camera. For the point reconstruction in CSEC, there is a new homography from the new external camera to the original external camera ahead of the H^D,A^ in Eq. (5). According to the chain rule of the partial derivatives, the extrinsic parameters, intrinsic parameters and image coordinates of the added camera can be investigated by the similar process of the partial derivatives of the parameters.

## Summary

The analysis models are built to determine the reconstruction accuracy of the spatial coordinates reconstructed by the active vision system with two cameras and a 3D orientation reference. The influences of the structure parameters on the reconstruction accuracy are analyzed in details. The factors are divided into three groups: extrinsic parameters of two cameras, intrinsic parameters of the internal camera and image coordinates of laser projection. The influences of the factors on the spatial point reconstruction are analyzed for the active vision system. Then the variations caused by the multiple parameters are analyzed in each group. The variation principles of the spatial coordinates are demonstrated for the vision system with two cameras and a 3D orientation reference. The research also provides a useful reference for the parameter selection of the application measurement fields and the calibration verification of the active vision system.

## Data Availability

The datasets generated during the current study are available from the corresponding author on reasonable request.
